# Neural Correlates of Cognitive Dysfunctions in Cervical Spondylotic Myelopathy Patients: A Resting-State fMRI Study

**DOI:** 10.3389/fneur.2020.596795

**Published:** 2020-12-23

**Authors:** Rui Zhao, Qian Su, Zhao Chen, Haoran Sun, Meng Liang, Yuan Xue

**Affiliations:** ^1^Department of Orthopedics Surgery, Tianjin Medical University General Hospital, Tianjin, China; ^2^Department of Molecular Imaging and Nuclear Medicine, National Clinical Research Center for Cancer, Tianjin Key Laboratory of Cancer Prevention and Therapy, Tianjin's Clinical Research Center for China, Tianjin Medical University Cancer Institute and Hospital, Tianjin, China; ^3^Department of Radiology, Tianjin Medical University General Hospital, Tianjin, China; ^4^School of Medical Imaging, Tianjin Medical University, Tianjin, China; ^5^Tianjin Key Laboratory of Spine and Spinal Cord, Tianjin Medical University General Hospital, Tianjin, China

**Keywords:** resting-state fMRI, cervical spondylotic myelopathy, cognitive deficits, BOLD variability, default mode network

## Abstract

Cervical spondylotic myelopathy (CSM) is a common disease of the elderly that is characterized by gait instability, sensorimotor deficits, etc. Recurrent symptoms including memory loss, poor attention, etc. have also been reported in recent studies. However, these have been rarely investigated in CSM patients. To investigate the cognitive deficits and their correlation with brain functional alterations, we conducted resting-state fMRI (rs-fMRI) signal variability. This is a novel indicator in the neuroimaging field for assessing the regional neural activity in CSM patients. Further, to explore the network changes in patients, functional connectivity (FC) and graph theory analyses were performed. Compared with the controls, the signal variabilities were significantly lower in the widespread brain regions especially at the default mode network (DMN), visual network, and somatosensory network. The altered inferior parietal lobule signal variability positively correlated with the cognitive function level. Moreover, the FC and the global efficiency of DMN increased in patients with CSM and positively correlated with the cognitive function level. According to the study results, (1) the cervical spondylotic myelopathy patients exhibited regional neural impairments, which correlated with the severity of cognitive deficits in the DMN brain regions, and (2) the increased FC and global efficiency of DMN can compensate for the regional impairment.

## Introduction

Cervical spondylotic myelopathy (CSM) is commonly observed in clinical practice, and its severity depends on the pathophysiological effects on the spinal cord (1–3). However, emerging evidence suggest that cortical alterations may also be crucial in CSM pathology (4–7). It has been shown that the cortical alterations can only partly restore after decompression surgery (8, 9), and the preoperative neural activities can be used as a potential biomarker to predict surgical outcomes (10). To investigate functional alteration at the brain level in CSM patients, functional magnetic resonance imaging (fMRI), which is a highly effective method for investigating neurological and psychological conditions, has been widely applied (11–15). This technique measures blood-oxygenation-level-dependent (BOLD) signals and is used to study brain functional alterations in diseases like Alzheimer's disease (AD), Parkinson's disease (PD), epilepsy, attention-deficit hyperactivity disorder (ADHD), and mood disorders (16–23). In neuroimaging studies of CSM, fMRI has illuminated functional alterations in motor cortices (4, 7, 10, 24, 25). Using resting-state fMRI (rs-fMRI), we have previously identified alterations in functional connectivity in visual cortices that correlate with visual and motor function in CSM patients (26, 27).

Further, recent studies have intermittently identified symptoms beyond the cord, including depression, anxiety, blurred vision, and cognitive deficits (9, 28–31). However, the mechanisms underlying CSM pathophysiology at cortical level are poorly studied, and the cognitive aspects are rarely studied in CSM patients. We are aware of only one published article reporting the use of single photon emission computed tomography (SPECT) to investigate neural mechanism of cognitive deficits in CSM patients (9). This study showed reduced regional cerebral blood flow (rCBF) in the posterior cortical areas, as well as blood perfusion in occipital and parietal lobule improvements after spinal cord decompression surgery. Moreover, language, cognitive, and visual deficits were partially restored. However, that study did not quantifiably correlate rCBF change with the cognitive function level. Additionally, SPECT cannot evaluate instantaneous brain signals that directly correlate with fluctuating neuronal activities and is not amenable to network analysis.

Here, we investigated the neural mechanisms of cognitive deficits in CSM patients. We used Montreal cognitive assessment (MoCA) to evaluate cognitive function in CSM patients. To assess brain neural activity, we calculated the BOLD signal variability, which is an established method of quantifiably evaluating BOLD level fluctuations (32–36). Moreover, BOLD variability has been shown to provide brain functional architecture in aging adults and the cognitive declined diseases (21–23, 37–41). We used functional connectivity analysis and graph theoretical analysis to better investigate brain network changes in CSM patients. We hypothesized that BOLD signal variability and network alterations in CSM patients correlate with impaired cognitive function.

## Materials and Methods

### Subjects

Ethical approval for this study was granted by the institutional review board of Tianjin Medical University General Hospital (Tianjin, China). All participants gave written informed consents.

#### Dataset1

Twenty-seven right-handed CSM patients were recruited from 2015 to 2016 using the following inclusion criteria: (a) clear MRI evidence of cord compression on the cervical spine; (b) clinical manifestations of sensorimotor extremity deficits or bladder/bowel dysfunction; (c) participants agreed to undergo decompression of the spinal canal; (d) no history of cervical spinal surgery; (e) ability to complete fMRI studies; (f) no stenosis of the extracranial vertebral artery and the carotid artery after Doppler ultrasound examination; (g) no clinical evidence or history of other neurological, psychiatric, ocular disease, or systemic diseases like hypertension and diabetes; and (h) no history of alcohol and substance abuse. Eleven healthy subjects of similar age, gender, and education were recruited through advertisements with the following inclusion criteria: (a) no evidence of spinal compression, (b) no ocular disease, (c) no other spinal or brain neurological disorders, or systemic disease, and (d) ability to complete fMRI studies. We have previously reported detailed participants' information (27).

#### Dataset2

Twenty-six CSM patients and 36 healthy controls were recruited in 2019–2020 using the aforementioned inclusion criteria. Thus, the current study had a total of 53 CSM patients and 57 healthy participants.

### MR Data Acquisition and Preprocessing

#### Dataset1

Data were acquired using a 3.0-T magnetic resonance scanner (Discovery MR750, General Electric) with an eight-channel phased-array head coil. Before scanning, earplugs were used to minimize noise. Participants were instructed to keep their heads still during scanning, and a sponge pad was used to support the head and minimize unconscious movement. Participants were also instructed to keep their eyes closed but remain awake while avoiding specific and strong thoughts. Functional images were collected using gradient echo-planar pulse imaging (EPI) sequence using the following parameters: repetition time = 2,000 ms, echo time = 30 ms, flip angle = 90°, field of view = 240 × 240 mm, matrix = 64 × 64, 38 slices, slice thickness = 3.0 mm. One hundred eighty images were collected in 6 min. Structural images were collected using a 3D T1-weighted image (3D T1WI) for coregistration and normalization of functional images with the following parameters: sagittal acquisition; repetition time = 7.8 ms; echo time = 3.0 ms; inversion time = 450 ms; flip angle = 13°; field of view = 256 × 256 mm; matrix = 256 × 256; 180 slices; slice thickness = 1.0 mm.

#### Dataset2

Three-Tesla fMRI data were acquired using a MAGNETOM Prisma 3 T MR scanner (Siemens, Erlangen, Germany) with a 64-channel phase-array head–neck coil. Participants were instructed to keep their heads still during scanning, and a sponge pad was used to support the head and minimize unconscious movement. Participants were also instructed to keep their eyes closed but remain awake while avoiding specific and strong thoughts. BOLD signals were collected using prototype simultaneous multislices gradient echo EPI sequence using the following parameters: echo time (TE) = 30 ms; repetition time (TR) = 800 ms, field of view (FOV) = 222 × 222 mm; matrix = 74 × 74; in-plane resolution = 3 × 3 mm; flip angle (FA) = 54°; slice thickness = 3 mm; gap = 0 mm; number of slices = 48; slice orientation = transversal; bandwidth = 1,690 Hz/pixel, parallel acquisition technique (PAT) mode; slice acceleration factor = 4; phase encoding acceleration factor = 2. Four hundred fifty images were taken in 6 min. A high-resolution 3D T1 structural image (two inversion contrast magnetization prepared rapid gradient echo sequence, MP2RAGE) was also acquired using the following parameters: TR/TE = 4,000 ms/3.41 ms; inversion times (TI1/TI2) = 700 ms/2,110 ms; FA1/FA2 = 4°/5°; matrix = 256 × 240, FOV = 256 × 240 mm; number of slices = 192; in-plane resolution = 1 × 1 mm; slice thickness = 1 mm; slice orientation = sagittal; total duration is 6 min, 42 s.

All MR data were preprocessed using the toolbox Data Processing Assistant for rs-fMRI (DPARSF; http://www.restfmri.net/forum/DPARSF) pipeline. Briefly, 180 volumes were acquired for functional scanning in dataset1, and 450 volumes were acquired for functional scanning in dataset2. The first 10 volumes of each functional scans corresponding with participants acclimatization to the scanning environment and magnetization stabilization were excluded. Slice-timing correction and motion correction were performed to remove timing differences and head movement, respectively (due to significantly shortened TR, slice-timing correction was not applied to dataset2). Functional images were coregistered to structural images and spatially normalized to the Montreal Neurological Institute template. Each voxel was resampled to 3 × 3 × 3 mm^3^. The liner-drift, Friston-24 parameters, the mean global signal, the white matter signal, and cereberospinal fluid (CSF) signal were extracted as covariates and regressed out to minimize non-neural signals. Subsequently, scrubbing step for high motion timepoints was also performed. Finally, a bandpass filter (0.01–0.08 Hz) was then applied to remove high-frequency noise effects and then smoothed with a 5-mm full-width-half-maximum isotropic Gaussian kernel. Resulting data were used for further analysis.

### Cognitive Function Assessment

MoCA was applied to evaluate cognitive function. Fifteen, and all patients, completed behavior scaling in dataset1 and dataset2, respectively. All patients were fully examined by an experienced orthopedist using the Japanese Orthopedic Association (JOA) scale evaluation.

### Analysis of BOLD Signal Variability

Using the preprocessed functional images in standard space, signal variability was calculated in a voxel-wise fashion using the std command to calculate signal standard deviation on MATLAB R2017a. For each participant, a single value was generated for each brain voxel, representing the standard deviation (SD) across the whole functional scan. To reveal differences between CSM patients and HC, two-sample *t*-test was performed within the gray matter masks using age, gender, scan parameters, and education as covariates. *p* ≤ 0.001 (significance threshold) was corrected for multiple comparisons with familywise error correction at the cluster level, corresponding to a corrected *p* ≤ 0.05, using SPM12 (http://www.fil.ion.ucl.ac.uk/spm). Peak coordinates of the clusters, which showed significant differences between groups, were selected and used as centers to generate 8-mm spheres as regions of interest (ROI). Next, BOLD signal SDs were extracted from the ROIs and correlation analysis done to detect the relationship between impaired BOLD signal variabilities and behavior scales.

### Seed-Based Functional Connectivity Analysis

To evaluate the functional changes between impaired regions and the distant brain regions in CSM patients, seed-based functional connectivity analyses were done. To this end, the ROIs selected in section Analysis of BOLD Signal Variability were used for seed-based functional connectivity analyses. Briefly, for each sphere, mean time series was extracted and Pearson correlation coefficient with each voxel's time series calculated, generating a voxel-wise FC map for each participant. Next, all R values were transformed to Z-scores using Fisher z-transformation. Two-sample *t*-tests were then conducted for gray matter masks, using age, gender, scan parameters, and education as covariates. A significance threshold of *p* ≤ 0.001 was used to correct for multiple comparisons with familywise error correction at the cluster level. The resulting corrected *p*-value was < 0.05). Correlation analyses in CSM patients within the clusters, which was used to evaluate the relationship between FC changes and behavior scores revealed significant between-group differences. False discovery rate correction (FDR) was performed to correct resultant *p*-values for multiple comparison correction.

### Functional Network Analysis and Graph Theoretical Analysis

DMN functional impairments have been observed in various contexts, including neurological and neuropsychiatric disorders, and aging. Our analyses in sections Analysis of BOLD Signal Variability and Seed-Based Functional Connectivity Analysis found that DMN exhibits regional functional impairment in CSM patients. However, global DMN changes have not been previously investigated. For network analysis, nodes were first defined using Power templates (264 nodes) (42). Given that our study only tended to evaluate DMN function, only the 58 nodes within DMN were extracted. Next, meantime series were extracted from each node. Pearson correlations of the mean time series between all node pairs within DMN were considered edges of the brain functional network (58 × 58). Two-sample *t*-tests were performed to reveal the edges' differences between CSM patients and healthy controls (HCs) with age, gender, scan parameters, and education as covariates. Significance cutoff was *p* = < 0.05/(58 × 57/2) (Bonferroni correction).

Graph theoretical analyses can reveal the topological aspects of brain functional and structural networks in healthy adults and patients. In our study, a thresholded (z > 0.5) and binarized individual connectivity matrix (264 × 264) was conducted for each subject. Due to the potential functional segregation of network nodes, we did not use a proportional thresholding approach [detailed discussion can be found in (43)]. We estimated global network parameters, including shortest path (average across all nodes), assortativity, global efficiency (normalized by dividing the mean of 100 random networks' global efficiency), hierarchy, and synchronization. Regional nodal parameters included nodal degree, nodal efficiency, and nodal betweenness (44). Two-sample *t*-tests were used to detect network global and nodal differences between CSM patients and healthy controls using age, gender, scan parameters, and education as covariates. Resulting *p*-values were subjected to false discovery rate correction. All graphic theoretical analyses were calculated using GRETNA, a publicly available MATLAB toolbox (45).

### Validation Analysis

To analyze the potential influence of different number of timepoints between two independent datasets. Dataset2 was down-sampled to balance this effect. Three approaches have been adopted to down-sample dataset2: (1) pick out one timepoint out of every two timepoints, resulting in 220 timepoints after down-sampling procedure; (2) pick out one timepoint out of every three timepoints, resulting in 147 timepoints after down-sampling procedure; and (3) pick out one timepoint out of every four timepoints, resulting in 110 timepoints after down-sampling procedure. Subsequently, two-sample *t*-tests were performed (i.e., as described in section Analysis of BOLD Signal Variability) and were corrected for multiple comparisons with familywise error correction at the cluster level. The resultant T maps were then binarized, and the dice coefficients among these binary masks were calculated to measure the level of overlap between two binary masks. Besides, all analyses in sections Analysis of BOLD Signal Variability, Seed-Based Functional Connectivity Analysis, and Functional Network Analysis and Graph Theoretical Analysis were performed using the down-sampled data (i.e., down-sampling dataset2 to 220 timepoints, dataset1 remain the same).

To analyze the potential influence of head motion in this study, framewise displacement (FD) values, which quantifiably estimate head motion during scan, were calculated, averaged across all timepoints in all participants, and compared between groups. The FD value was calculated using three robust methods, the Jenkinson method (46), Power method (47), and VanDijk method (48). We also performed voxel-wise correlation analyses between mean FD values and signal variability across CSM patients, across HC patients, and across all participants. Besides, we also performed region-wise correlation analyses within brain regions exhibited significant group differences in section Analysis of BOLD Signal Variability. The mean signal variability within these regions were extracted and correlated with the mean FD values. Moreover, we reperformed in section Analysis of BOLD Signal Variability by adding the max FD Jenkinson, mean FD Jenkinson, median FD Jenkinson values, and six head motion parameters as covariates.

To further test the consistency of the results across datasets in our current study, we first compared the voxel-wise signal variability between groups in each dataset separately; then, the resultant T maps were corrected by family-wise error (FWE) correction and binarized to calculated the dice coefficient between two binarized masks. Subsequently, the same procedure was performed to test the consistency of the FC results between datasets; however, we found that no significant difference was observed within each dataset due to the relative low sample size of dataset and the strict multiple comparison correction method. We, therefore, only compared the results between datasets within brain regions reported in **Table 4**. We first extracted the FCs within these brain regions and two-sample *t*-tests were performed between CSM and HCs in each dataset separately.

To explore the potential relationship between altered FC and altered signal variability, correlation analyses were performed.

## Results

### Demographic Data

Demographic data and behavior-scale scores, including MoCA, Mini-Mental State Examination (MMSE), and JOA are shown in Table 1. There were no significant intergroup differences with regards to age, gender, or education years (*p* ≤ 0.05).

**Table 1:** Demographic data of the two datasets.

	**Dataset1**			**Dataset2**		
	**CSM (*n* = 27)**	**HC (*n* = 11)**	***p*-value**	**CSM (*n* = 26)**	**HC (*n* = 36)**	***p*-value**
Age (years)	57.9 ± 9.1	54.8 ± 8.4	0.34	54.7 ± 8.8	53.7 ± 8.3	0.54
Gender (F/M)	12/15	5/6	0.96	12/14	17/19	0.93
Education (years)	10.8 ± 2.7	11.6 ± 2.5	0.42	10.7 ± 2.5	11.2 ± 23	0.41
JOA	11.8 ± 1.5			11.0 ± 1.8		
MoCA	23.3 ± 3.3 (*n* = 15)			23.2 ± 2.5		
MMSE	25.1 ± 2.1 (*n* = 15)			23.2 ± 1.8		

### Reduced BOLD Signal Variability in CSM Patients Correlates With Impaired Cognitive Function

**Figure 1:**
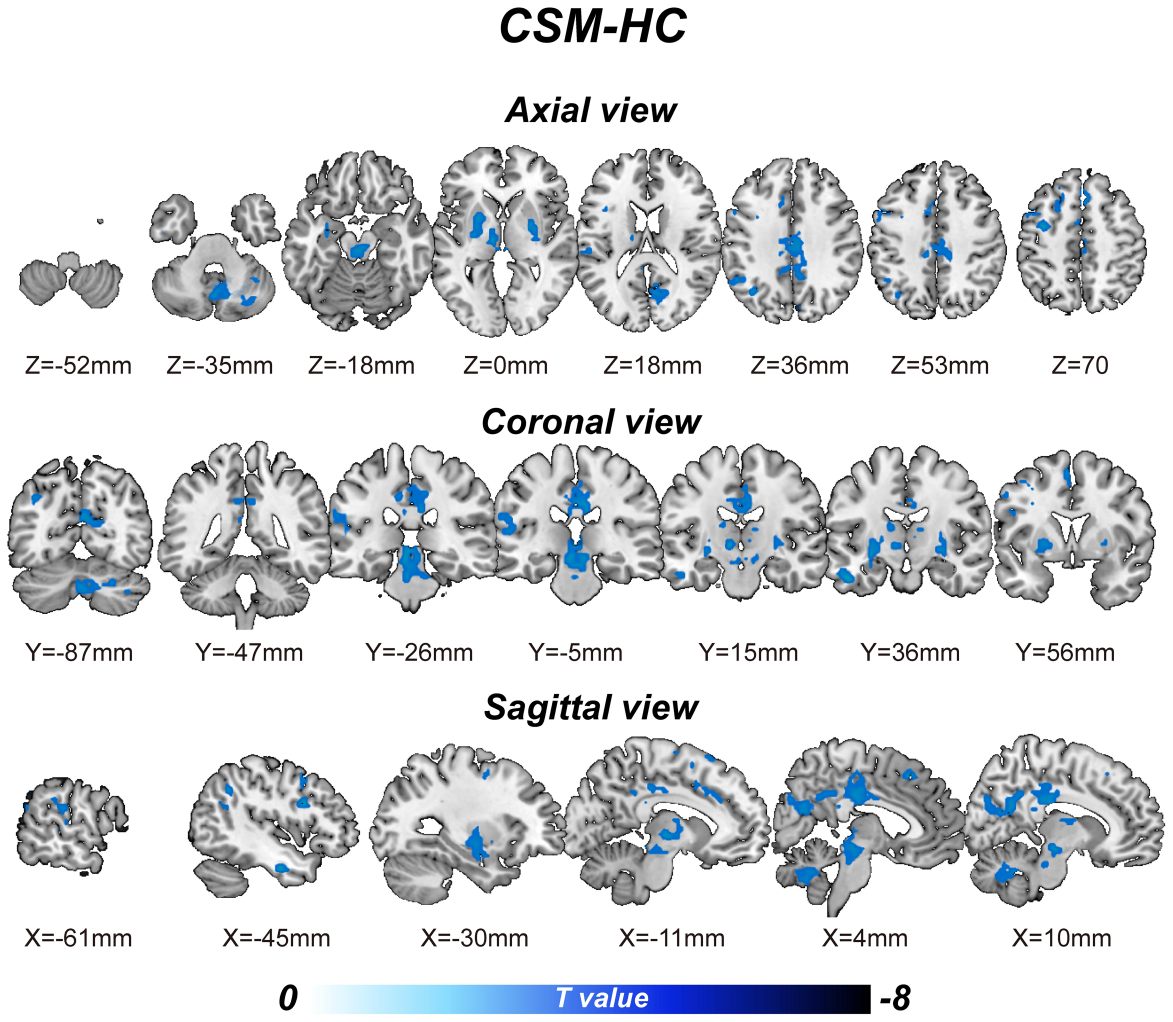
The voxel-wise blood-oxygenation-level-dependent (BOLD) signal variability [defined via the signal's standard deviation (SD)] differences between the cervical spondylotic myelopathy (CSM) patients and the healthy controls (HCs).

Relative to the control group, CSM patients exhibited significantly lower BOLD-signal variability [*p* ≤ 0.001 (familywise error correction), corrected *p* ≤ 0.05 at cluster level; Table 2] in various brain regions, including the right cerebellum posterior lobe, left inferior temporal gyrus, left thalamus, mid brain, left putamen, left pallidum, right putamen, right pallidum, left supramarginal gyrus, left superior temporal gyrus, bilateral middle cingulate gyrus, right precuneus, right cuneus, right calcarine gyrus, bilateral posterior cingulate gyrus, left precentral gyrus, left inferior frontal gyrus, left angular gyrus, left inferior parietal gyrus, left superior frontal gyrus, and bilateral supplementary motor area (Figure 1). Cluster peak coordinates were used to generate 8-mm spheres and the average variability within spheres extracted. Our analysis illustrated significant correlation between altered SD within spheres and the behavior scores measured. The variability extracted from the left inferior parietal lobule sphere had a significant positive correlation with MoCA scores (R = 0.351, *p* = 0.023; uncorrected). The variability extracted from the right mid-cingulate gyrus/the left precentral gyrus spheres exhibited significant positive correlation with JOA scores (R = 0.347, *p* = 0.026; and R = 0.353, *p* = 0.021, respectively; uncorrected). Correlation coefficients between behavior-scale scores and BOLD signal variabilities within predefined 8-mm peak group difference spheres are shown on Table 3.

**Table 2:** Regions of significant blood-oxygenation-level-dependent (BOLD) signal variability difference between cervical spondylotic myelopathy (CSM) patients and healthy controls (HCs).

**Brain regions**	**Peak MNI (x, y, z)**			**Voxels**	***t* value**
L precentral gyrus	−36	−3	48	122	−5.18
L inferior temporal gyrus	−48	−9	−27	47	−5.17
R putamen/R pallidum	27	−6	−3	65	−4.95
L thalamus	−6	−27	−12	282	−4.86
L putamen/L pallidum	−27	−6	−6	123	−4.77
R precuneus/R calcarine/B posterior cingulate gyrus	6	−24	33	443	−4.69
L angular gyrus	−45	−54	39	91	−4.64
R cerebellum posterior lobe	−3	−69	−33	188	−4.54
L supramarginal gyrus/L superior temporal gyrus	−54	−27	18	49	−4.29
B supplementary motor area	0	27	48	68	−4.29

*The clusters exhibited a significant difference between-group in CSM patients and HCs. L, left; R, right; B, bilateral*.

**Table 3:** The correlation coefficients between the behavior scale scores and the blood-oxygenation-level-dependent (BOLD) signal variabilities within 8-mm peak group difference spheres predefining.

	**MMSE**	**MoCA**	**JOA**
R cerebellum	−0.01	0.13	0.03
L inferior temporal gyrus	−0.19	−0.19	0.16
L thalamus	−0.29	0.26	−0.04
L putamen	−0.01	0.12	0.05
R putamen	–0.33*	0.19	0.02
L superior temporal gyrus	0.06	0.13	0.01
R middle cingulate gyrus	−0.26	−0.06	0.34*
L precentral gyrus	0.05	−0.06	0.35*
L inferior parietal lobule	0.09	0.35*	0.04
L superior frontal gyrus	−0.06	−0.16	−0.05

* *p < 0.05 (uncorrected), the significant correlation coefficients were shown in bold*.

### Voxel-Wise Functional Connectivity Map Difference Between CSM Patients and Healthy Controls

Next, spheres generated in section Reduced BOLD Signal Variability in CSM Patients Correlates with Impaired Cognitive Function were seeded into whole brain FC analysis. Two-sample *t*-test analyses revealed differences between CSM patients and HCs. We find that all FCs, which exhibited significant group difference (*p* ≤ 0.001, familywise error correction, corrected *p* ≤ 0.05 at cluster level, Table 4), increased in CSM patients. Altered FCs include connectivity between the left posterior cerebellum lobe and left superior frontal gyrus, connectivity between the left posterior cerebellum lobe and left mid-posterior cerebellum, connectivity between the left inferior temporal gyrus and left posterior cerebellum lobe, connectivity between the left inferior temporal gyrus and right fusiform gyrus, connectivity between the left inferior temporal gyrus and bilateral calcarine gyrus, connectivity between the left inferior temporal gyrus and right inferior occipital gyrus, connectivity the between left superior temporal gyrus and left middle frontal gyrus, connectivity between the left precentral gyrus and right calcarine gyrus, connectivity between the left inferior parietal gyrus and bilateral calcarine gyrus, and connectivity between the left inferior parietal gyrus and right superior occipital gyrus.

**Table 4:** Regions of significant functional connectivity differences between cervical spondylotic myelopathy (CSM) patients and healthy controls (HCs).

**Seeds**	**Regions**	**Peak MNI**			**Voxels**	**T**
R posterior cerebellum lobe	R cerebellum posterior lobe	24	−84	−48	83	4.77
	R superior frontal gyrus	−12	63	24	233	4.98
L inferior temporal gyrus	L cerebellum posterior lobe	−39	−51	−45	71	4.75
	R fusiform gyrus	−21	−72	−24	55	4.42
	B calcarine	6	−90	21	583	5.46
	R inferior occipital gyrus/R middle occipital gyrus	−15	−99	−12	79	4.64
L superior temporal gyrus	L middle frontal gyrus	−42	54	6	76	4.39
L precentral gyrus	R calcarine/R inferior occipital gyrus	24	−102	−9	66	4.95
L inferior parietal lobule	B calcarine/R superior occipital gyrus	6	−90	21	777	5.74

*The clusters showed significant between-group functional connectivity differences between CSM patients and HCs*.

**Figure 2:**
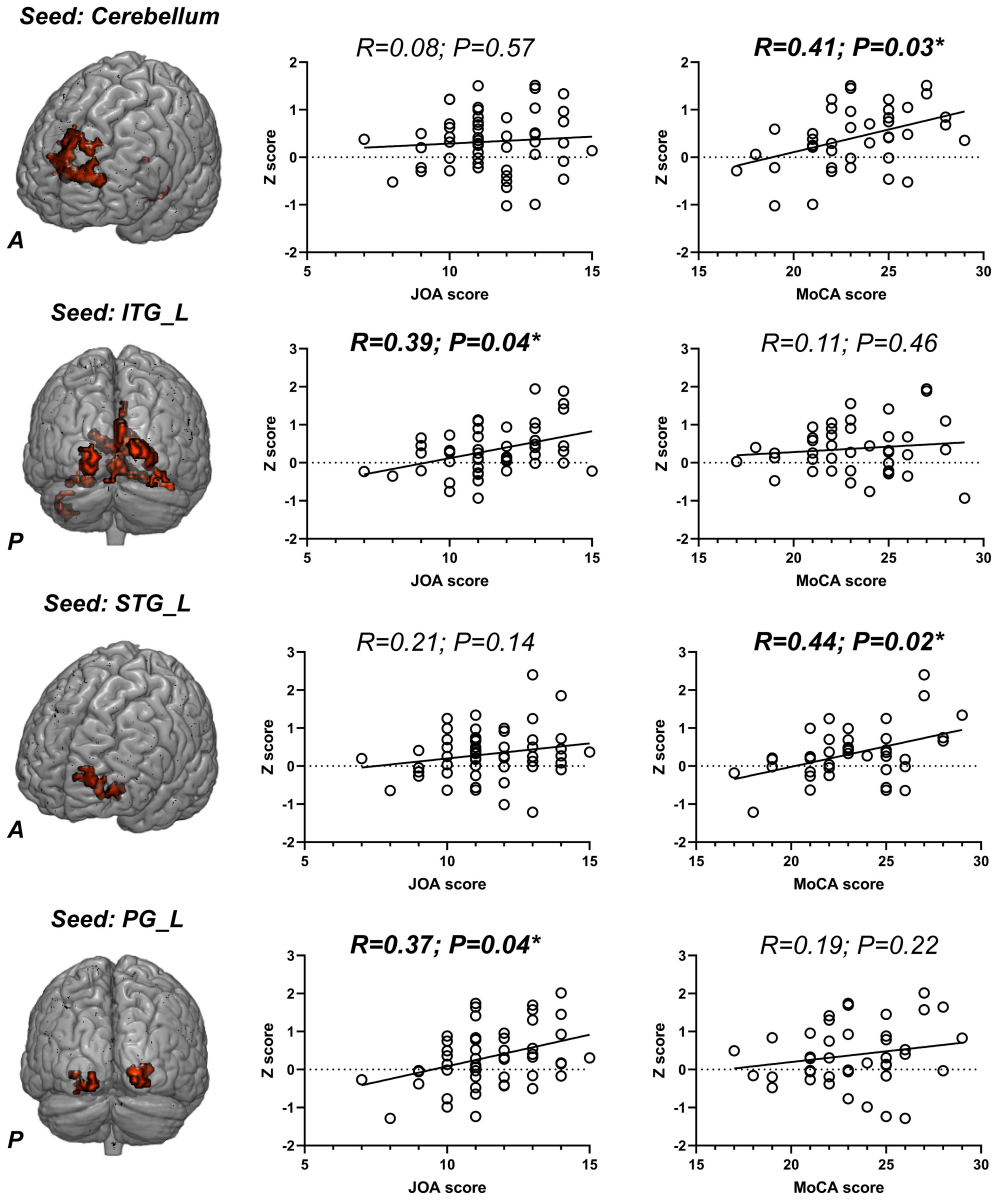
The seed-based functional connectivity (FC) differences between cervical spondylotic myelopathy (CSM) patients and healthy controls (HCs); the scatter plots of the correlation between altered FCs and Japanese Orthopedic Association (JOA)/Montreal cognitive assessment (MoCA) scores. ITG, inferior temporal gyrus; STG, superior temporal gyrus; PG, precentral gyrus; L, left; R, right. *p < 0.05 (FDR corrected) and the significant correlation coefficients and p-values were shown in bold.

Next, altered FCs in the aforementioned regions (Table 4) were extracted and correlation analyses between altered FCs and behavior-scale scores performed. This analysis revealed that the FC between the cerebellum and left superior frontal gyrus significantly correlated with MoCA scores (R = 0.41, *p* = 0.03; FDR corrected). FC between left posterior cerebellum lobe and left mid-posterior cerebellum exhibited significant positive correlation with MoCA scores (R = 0.31, *p* = 0.04; uncorrected). FC between the left inferior temporal gyrus and bilateral calcarine gyrus positively correlated with JOA scores (R = 0.39, *p* = 0.04; FDR corrected). FC between the left inferior temporal gyrus and left middle frontal gyrus positively correlated with MoCA scores (R = 0.44, *p* = 0.02; FDR corrected) and FC between the left precentral gyrus and right calcarine gyrus had a significant positive correlation with JOA scores (R = 0.37, *p* = 0.04; FDR corrected) (Figure 2).

### Functional DMN Connectivity Changes in CSM Patients

Region-wise functional connectivity analysis revealed increased FC between the superior frontal gyrus and anterior cingulate gyrus (two nodes within ACC) (*t* = 4.98, *p* = 2.71e−06; *t* = 4.91, *p* = 3.83e−06; survived Bonferroni correction).

### Global and Nodal DMN Changes in CSM Patients

**Figure 3:**
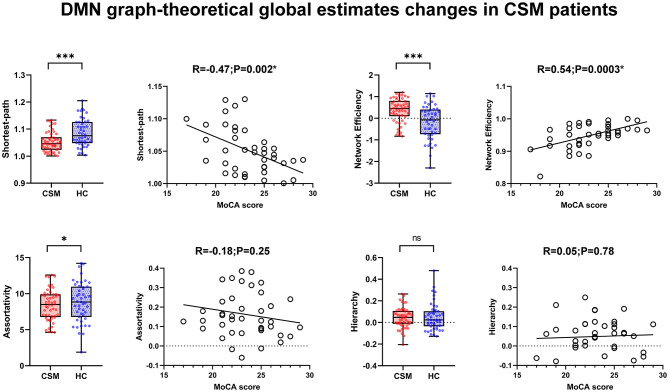
The global parameters change in cervical spondylotic myelopathy (CSM) patients, and the scatter plot of the correlation between altered global parameters and Montreal cognitive assessment (MoCA) scores. *In scatter plots means *p* < 0.05 (uncorrected) and *In box plots mean *p* < 0.05 (uncorrected), ***p < 0.001 (uncorrected).

Graphic theoretical analysis of global-wise parameters revealed decreased global shortest path (average across all nodes) (*t* = −3.36, *p* = 0.001), increased global efficiency (*t* = 3.69, *p* = 0.0004), and decreased assortativity (*t* = −2.43, *p* = 0.01) in CSM patients. MoCA scores indicated significant correlation with altered shortest path (R = −0.47, *p* = 0.002; uncorrected) and global efficiency (R = 0.54, *p* = 0.003; uncorrected), respectively. Significant intergroup hierarchy differences were detected (*t* = −0.45, *p* = 0.66). There was no significant correlation between MoCA scores and assortativity/hierarchy (R = −0.18, *P* = 0.25; R = 0.05, *p* = 0.78; uncorrected), respectively (Figure 3). We did not identify any nodal changes in CSM patients, with only few nodal parameters revealing small intergroup differences (Supplementary Figure 1).

### Validation Analyses

We found that the dice coefficients among all four T maps were all above 0.80, indicating a similar spatial distribution among them (see Supplementary Figure 1). The spatial distribution of each resultant T map was shown in Supplementary Figure 2. Therefore, it is unlikely that the effects we observed for the measured variability in the present study were due to differences in number of timepoints.

**Figure 4:**
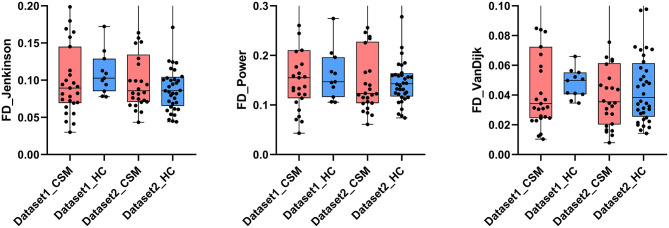
The framewise displacement (FD) value calculated via Jenkinson, Power, and VanDijk method as compared between the groups. No significant differences in FD values were observed. The corresponding mean values of the groups are shown at the bottom of each bar.

Given the potential influence of head motion on our data, we calculated framewise displacement (FD) to estimate motion in all subjects. This analysis did not identify significant differences in the FD values of patients vs. healthy controls, based on all three methods (Jenkinson method: dataset1, *p* = 0.70, *t* = 0.39; dataset2, *p* = 0.49, *t* = 0.70; Power method: dataset1, *p* = 0.67, *t* = 0.43; dataset2, *p* = 0.94, *t* = 0.07; VanDijk method: dataset1, *p* = 0.86, *t* = 0.19; dataset2, *p* = 0.81, *t* = 0.24). There were no significant FD values differences across datasets (Jenkinson method: patients, *p* = 0.77, *t* = 0.29; healthy controls, *p* = 0.48, *t* = 0.71; Power method: patients, *p* = 0.66, *t* = 0.44; healthy controls, *p* = 0.99, *t* = 0.001; VanDijk method: patients, *p* = 0.74, *t* = 0.33; healthy controls, *p* = 0.92, *t* = 0.10) (Figure 4).

We found that no significant correlation was observed by voxel- and region-wise analyses (see Supplementary Figures 4–7). We also performed two-sample *t*-test between two groups by adding FD Jenkinson (mean, max, and median) and motion parameters (six mean head motion parameters) to other covariates (age, gender, scan parameters, and education) as the review recommended. We found that despite a slight change in clusters' sizes and peak *t*-values, the majority of each cluster and the peak MNI coordinates of 7 out of 10 regions reported in Table 2 still remained the same or only moved by few voxels (see Supplementary Figure 8 and Supplementary Table 1).

**Figure 5:**
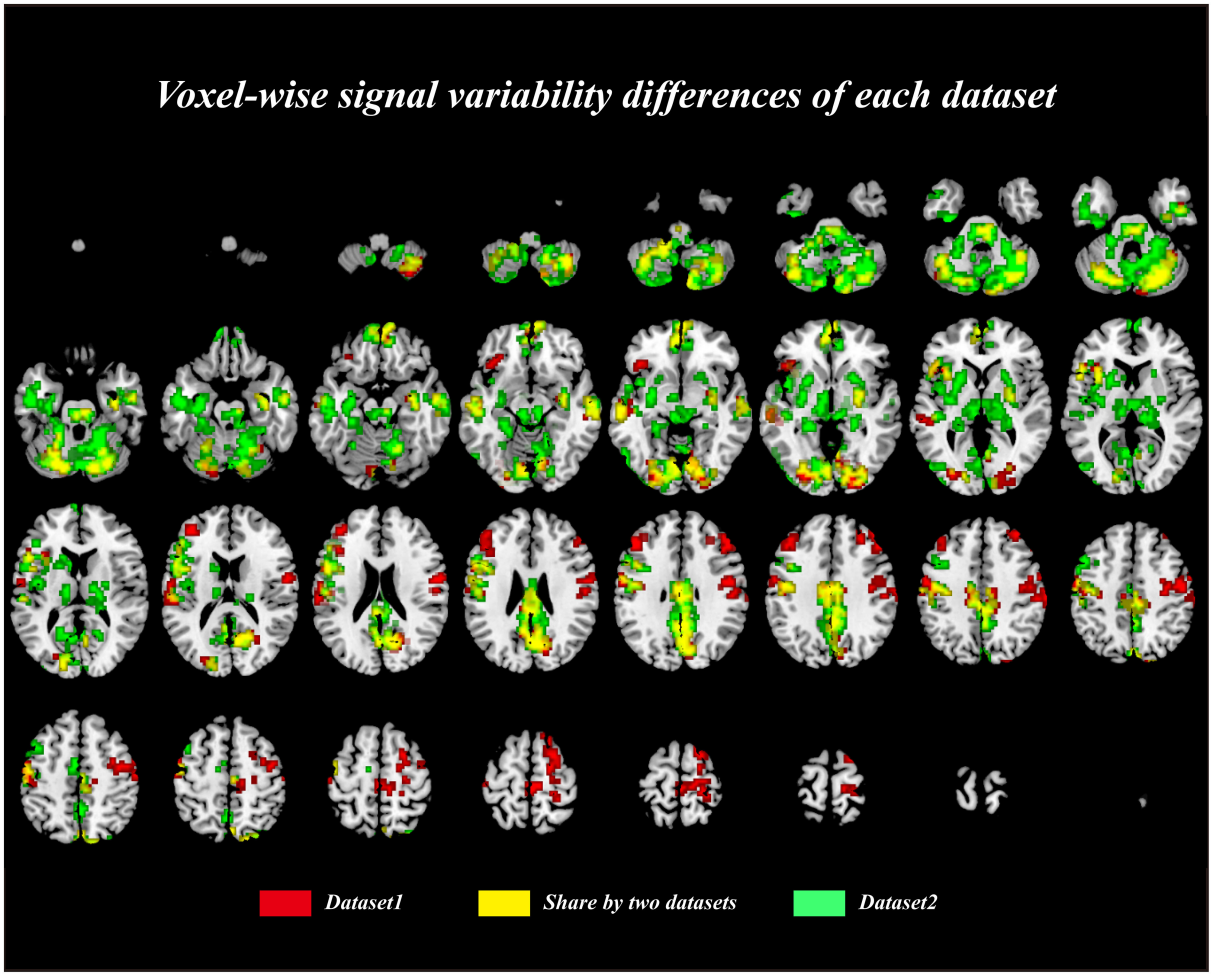
The voxel-wise signal variability differences between cervical spondylotic myelopathy (CSM) patients and healthy controls (HCs) in each dataset. The yellow color represented the overlapped brain regions across two datasets.

To further test the consistency of the results across datasets in our current study, we calculated the dice coefficient between two binarized results masks of each dataset. We found that the spatial distribution of these two masks were very similar, and the dice coefficient is 0.61, indicating a large proportion of the resultant clusters are overlapped (Figure 5). We noticed that there is discrepancy of the results between two datasets; therefore, we only focused on the results that consistent between two datasets. Subsequently, the same procedure was performed to test the consistency of the FC results between datasets; however, we found that no significant difference was observed within each dataset due to the relative low sample size of the dataset and the strict multiple comparison correction method. We, therefore, only compared the results between datasets within brain regions reported in Table 4. We first extracted the FCs within these brain regions, and two-sample *t*-tests were performed between CSM and HCs in each dataset separately (see Supplementary Figure 10). We found that these results were consistent between two datasets, indicating that the results reported in our main text are relatively stable.

We failed to detect any significant correlation between FC and signal variability in our current study.

More importantly, we found that the effects we observed for signal variability, functional connectivity, and network global estimates can be replicated using the down-sampled data (i.e., down-sampling dataset2 to 220 timepoints while dataset1 remain the same). The brain regions in which signal variability exhibited significant association with clinical measures in CSM patients remain the same as in section Reduced BOLD Signal Variability in CSM Patients Correlates with Impaired Cognitive Function (see Supplementary Table 2). No major change in the effects for the functional connectivity was observed after down-sampling dataset2 (see Supplementary Figure 11), and the peak coordinates of the clusters with significant group differences remain the same or only moved by few voxels (see Supplementary Table 3). The correlation between altered FC and MoCA/JOA scores were shown in Supplementary Figures 12, 13. It worth mentioning that the effects we observed for correlation in section Voxel-Wise Functional Connectivity Map Difference Between CSM Patients and Healthy Controls were partly replicated using the down-sampled data. For the graph theory analysis, the global estimates changes were consistent with our previous results in Global and Nodal DMN Changes in CSM Patients (see Supplementary Figures 14, 15).

## Discussion

### CSM Patients Lowered Regional BOLD-Signal Variability Within Default Mode Network, Correlating With Impaired Cognitive Behavior

In the past decades, the ratio of signal variability (i.e., the standard deviation of signal) and noise variability (i.e., the standard deviation of noise) was mainly applied in estimating signal-to-noise ratio. The variability of the signals from fMRI scanning has been overlooked and taken as measurement-related confounds (33). In contrast, based on the Lawrence Pinneo argument decades ago, the neural variability “is not merely noise” (49), and several neuroscience subdisciplines (cellular, systems, behavioral) have confirmed that the brain is inherently variable from time to time, no matter the functional measurement process at each level of the nervous system (32). Accordingly, numerous researchers conducted their study based on variance and found that the capacity to understand and predict several important phenomena can improve dramatically (50–52). Therefore, recently, a lot of studies have used the variability of neural signals (e.g., variance of signals or standard deviation of signals) as a powerful tool for investigating brain function in healthy adults and the sick (21, 41, 53, 54). They established that the variability of BOLD signals varies across the entire brain and lifespan, its physiological significance indicator (55). Besides, many studies have revealed that BOLD signals variability are potential biomarker in assessing cognitive function in aging adults and individuals with cognitive deficits (40, 54–57). Their results indicated reduced variability in certain brain regions correlated with declined cognitive function. Thus, we hypothesized that reduced variability is observed in certain brain networks and is associated with cognitive deficits in CSM patients. In this study, there was diminished BOLD signal variability in the precuneus, mid-prefrontal gyrus, posterior cingulate gyrus, inferior parietal lobe, and angular gyrus, the main regions of DMN. A reduced signal variability often implied a poor regional performance due to the fixed signal level and revealed low regional neural activity. Moreover, the BOLD signal variability in the inferior parietal and angular gyrus correlated with the cognitive function measured through the MoCA scale.

In neuroimaging, DMN is a large-scale brain network that has been extensively studied. A constellation of brain regions including the precuneus, posterior cingulate cortex (PCC), medial prefrontal cortex (MPFC), and medial, lateral, and inferior parietal cortex was associated with the DMN (58). Most neurological and neuropsychiatric disorders such as Alzheimer's disease (AD), Parkinson's disease (PD), epilepsy (especially temporal lobe epilepsy), attention-deficit hyperactivity disorder (ADHD), and mood disorders are associated with DMN functional changes (16, 19, 59–62). The clinical significance of the DMN is related to the potential roles of the DMN, including memory consolidation (63), working memory (64), emotion process (65), external and internal environments sampling (66), etc. To date, many studies have confirmed the significance of impaired function of DMN in the pathology of many neurodegenerative and neurocognitive impairment disorders such as PD, AD, depression, etc. Based on this, we speculated that (1) the CSM causes regional functional impairments in several regions of DMN, and (2) the functional impairments of certain brain regions within DMN can partly elucidate the cognitive deficits in CSM patients. Moreover, the possible mechanism has been stated in an animal model of spinal cord injury. Studies on rats found that cell apoptosis and death was due to neuronal loss and cognitive changes from posttraumatic brain inflammation. Interestingly, this occurred not only in the regional spinal cord but also in the ventricular superior cortex and hippocampus (67). In the present study, the decreased BOLD signal variability in DMN can also be explained by the low neural activities caused by neuronal loss.

### Increased Functional Connectivity and DMN Global Efficiency Can Compensate for the Cognitive Deficits in CSM Patients

In this study, voxel-wise FC analysis revealed that the impaired regions of DMN had an increased FC connectivity in several brain regions inside and outside the DMN including the cerebellum lobe, frontal gyrus, calcarine, occipital gyrus, etc. Moreover, the increased FC between the superior temporal gyrus and the middle frontal gyrus, as well as the increased FC between the cerebellum and the media frontal gyrus, positively correlated with the MoCA scores. A possible assumption is that, due to the regional impairments within DMN, both inside and outside DMN connections improved to compensate for the regional functional alterations within the DMN. Recent studies have confirmed that the increased FC between DMN and other brain regions represents a high coupling among brain networks, improving the cognitive performance. According to region-wise FC analysis, the FC within DMN increased in respect to MoCA scores. This further supports our previous assumption.

Furthermore, information depicting the DMN at both node and global levels was provided by graphic theoretical analysis. Herein, the disrupted DMN in CSM patients had decreased global shortest path, assortativity, and increased global efficiency. The global shortest path (also known as the characteristic path length) is an estimate parameter of transport and communication between nodes within a network. Due to the possible disconnect in the graph, an alternative approach based on the harmonic mean of geodesic lengths has been presented to describe the network information transportation and is regarded as the global efficiency of the network (68). In this work, we measured both parameters to evaluate the information communication within DMN. The results found that the DMN in CSM patients had reduced characteristic path length and increased global efficiency. Moreover, the altered parameters positively correlated with the MoCA sores. We hypothesized that the DMN upregulated the internal information transport to compensate for the decreased regional neural activities. Assortativity measures the similarity of connections in the graph according to the node degree. This means that the high-degree nodes are more likely to be connected to the high- than low-degree nodes in the assortative network. This topology parameter reveals the relationship degree between neighboring nodes within the network. Here, we found that CSM patients had a low DMN assortativity. This indicated that the DMN of CSM patients tend to first transfer information in regional subnetwork then to the whole network level. Moreover, there was obvious increased functional connectivity and efficiency and decreased regional activities. Many previous studies have found a similar phenomenon. Chen et al. found decreased amplitude of low-frequency fluctuation (ALFF) and regional homogeneity (REHO) value within primary visual cortices and increased functional connectivity within the visual network (26). Subsequently, the same group conducted seed-based functional connectivity analysis. They found that the functional connectivity within the visual network and the functional connectivity between secondary visual cortices and cerebellum were significantly increased in CSM patients (27). These results are consistent with previous studies, where there was decreased regional neural activities and enhanced connectivity (4, 7, 69). These intrinsic functional changes in the patients with CSM can be associated with functional reorganization and reflects the innate cortical plasticity in patients with CSM. Notably, the enhanced connectivity indicates the adaptive changes in CSM patients. However, it should be mentioned that we can only observed a trend of negative correlation between altered FC and altered signal variability. Therefore, despite the fact that extensive literature has shown similar phenomenon, we can only provide a relative weak evidence for the speculation.

Moreover, the effects we observed for the increased FC in CSM patients may seem surprising at the first glance. However, this phenomenon (i.e., increased FC and increased network efficiency in CSM patients) has been continually reported in numerous neuroimaging studies of CSM (4, 10, 26, 70). These studies continuously reported increased FC in CSM patients, and this phenomenon has been interpreted as a compensatory or adaptive change due to abnormal ascending or descending signal caused by long-term spinal compression. It is believed that the increased FC indicated a more interconnected stage among brain regions, and these results are consistent with electroencephalography studies demonstrating larger cortical networks involved in motor volition in patients with chronic spinal cord injury compared with healthy controls, further supporting the hypothesis that there is a need for increased brain plasticity in patients with CSM in order to overcome functional deficits. Moreover, numerous clinical evidences could also provide some support for this hypothesis (71–74). It has been shown that patients who had undergone surgical removal of one hemisphere exhibited an increased intrahemispheric FC and network efficiency in the remaining hemisphere. Moreover, these patients (i.e., underwent hemispherectomy in childhood) retained surprisingly high levels of cognitive and sensorimotor abilities. In sum, these studies provide evidence on the neural reorganization that produces compensated cognition after the surgical removal of one hemisphere. However, it should be mentioned that despite the fact that the patients with hemispherectomy retained high levels of behavior abilities, their cognitive and sensorimotor abilities were still poorer than healthy participants. Similar to our finding, patients with hemispherectomy (i.e., poorer cognition) exhibited better network measures (e.g., higher FC and higher efficiency), while controls show lower network efficiency and FCs. This maybe because these increased network measures were presentations of cortical reorganization.

### Visual Network Alterations

Previously, decreased neural activities have been confirmed and correlated with the impaired visual acuity in the visual cortex of the CSM patients. In the current study, the CSM patients group exhibited a lower BOLD signal variability than the control group in the calcarine gyrus, precuneus, and mid-/inferior occipital gyrus at the primary and secondary visual cortex. The calcarine gyrus, the primary visual cortex, is a major detection site for direct visual information (75). The precuneus cortices and posterior parietal gyrus in the BA18 and BA19, recognized as the association visual area, receive neural signals from the primary visual cortex (76). They act as a visual information integration site and generate conscious perceptions. To date, several studies have indicated that the diminished BOLD responses within the visual cortex are inherent in most diseases such as open-angle glaucoma, anisometropic amblyopic, and optic neuritis (16, 77, 78). Here, we found diminished BOLD signal variability in CSM patients, suggesting impaired visual cortices. Further, we confirmed that the visual cortices had low neural activity in the CSM group. This indicates that distant spinal cord dysfunction influences brain function.

### Sensorimotor Network Alterations

In this study, there was decreased BOLD signal variability in the left M1, bilateral thalamus, and supplement motor area of CSM patients. The altered BOLD variability correlated with the JOA score. The functional impairment of M1 in CSM patients has been reported by Tan et al. (79). Besides, other neuroimaging studies related to CSM and reports were similar to previous spinal cord injury studies (80–83). The M1 is a key region in the sensorimotor network (SN) involved in many functions including planning, control, and execution of voluntary movements from and to downstream spinal cord, thus innervated muscle movements (84, 85). The CSM patients in this study presented with low neural activity in precentral gyrus, which can be associated with motor deficits.

## Limitations

To the best of our knowledge, the present study is the first to investigate the cognitive change in CSM patients via rs-fMRI. However, this study has several limitations. First, the postoperative data were not collected due to the possible artifacts and MRI heating of implants. Normally, the data collected after spinal cord decompression is crucial for verifying the cognitive change in CSM patients. The postoperative data collection requires long-term follow-up. This will be completed in the future since it is safe. Second, more comprehensive demographic, clinical, and behavioral assessments should be done in the future to systematically investigate the cognitive change in CSM patients by combining functional and structural MR data. Third, spinal cord MR data including DTI, DSI, and functional scan should be collected in the future to further elucidate the neural pathophysiology mechanism of cognitive impairment in CSM patients. Our study was limited to participants with cervical spondylotic myelopathy. Other control groups such as motor neuron diseases and neuromyelitis optical should be collected in the future to investigate the specific brain functional variability in CSM patients. Besides, the current study cannot fully rule out the potential influence of head motion, and new strategies of reducing the effect of motion are required in the future. In our current study, we only observed weak correlation between altered signal variability and behavior measures, and these correlations could not survive multiple comparison correction. Therefore, these results could only provide a weak evidence to support our speculation. Larger sample size would be needed for the future studies.

## Conclusion

This study results suggest that (1) the cervical spondylotic myelopathy patients have regional neural impairments, which are correlated with cognitive deficits, in widespread brain regions primarily located in DNN, and (2) the increased DNN functional connectivity and global efficiency can compensate the regional impairments.

## Data Availability Statement

The raw data supporting the conclusions of this article will be made available by the authors, without undue reservation.

## Ethics Statement

The studies involving human participants were reviewed and approved by Tianjin Medical University General Hospital. The patients/participants provided their written informed consent to participate in this study.

## Author Contributions

YX and ML designed the study and revised this article. HS, ZC, and RZ collected the data. RZ and QS analyzed the data and wrote this article. All authors contributed to the article and approved the submitted version.

## Conflict of Interest

The authors declare that the research was conducted in the absence of any commercial or financial relationships that could be construed as a potential conflict of interest.
