# Supplementary material for: Neural Correlates of Cognitive Dysfunctions in Cervical Spondylotic Myelopathy Patients: A Resting-State fMRI Study
Source: Front Neurol. 2020 Dec 23;11:596795. doi: 10.3389/fneur.2020.596795 (PMC7785814; doi:10.3389/fneur.2020.596795)
Supplement: Supplementary file 1 [file Table_1.DOCX]

**Sup-Figure 1.**


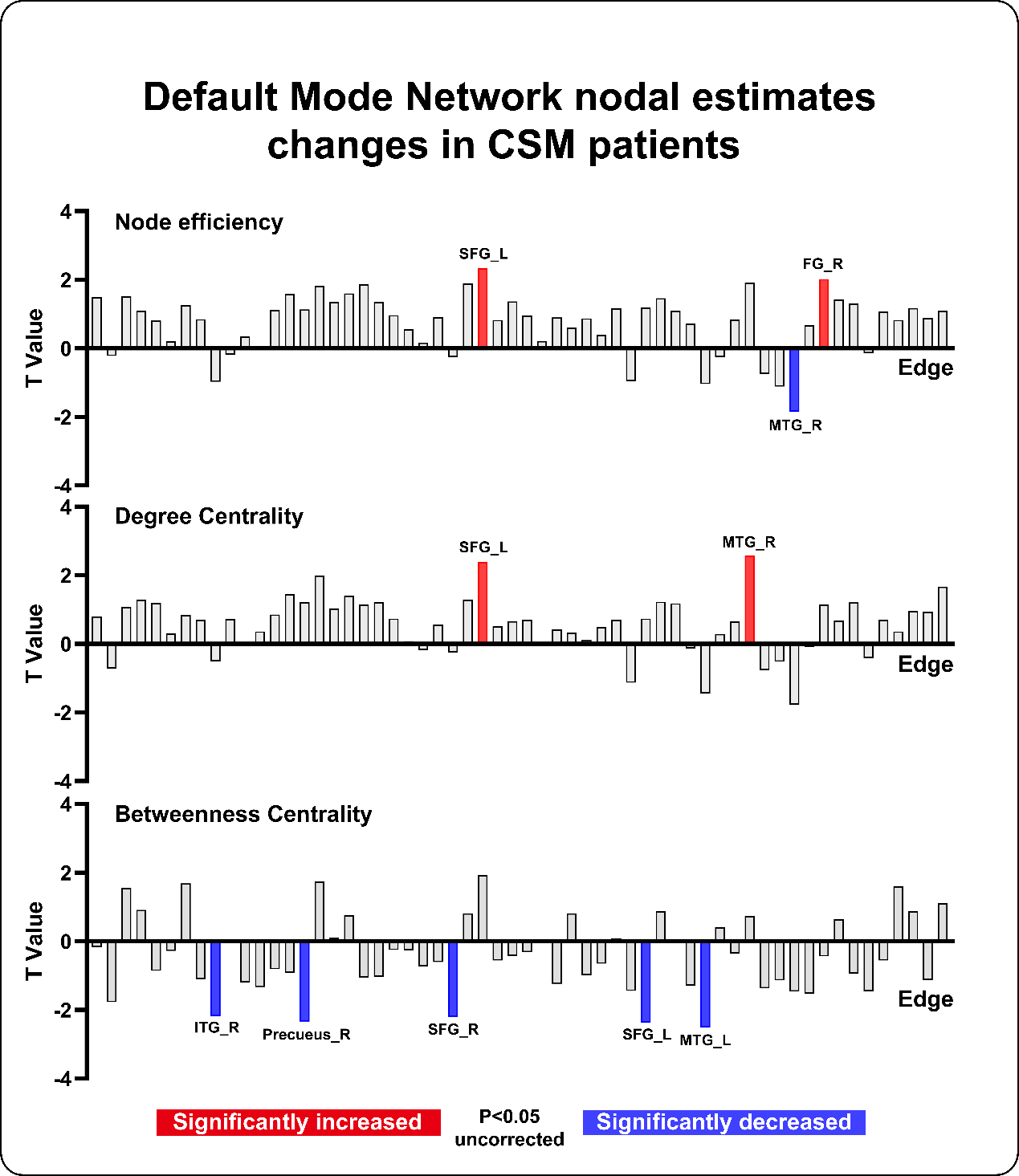


The default mode network nodal parameters difference between Cervical Spondylotic myelopathy (CSM) patients and Healthy Controls (HCs). SFG: superior frontal gyrus; MTG: middle temporal gyrus; FG: fusiform gyrus; ITG: inferior temporal gyrus; L: left; R: right.

**Sup-Figure 2.**


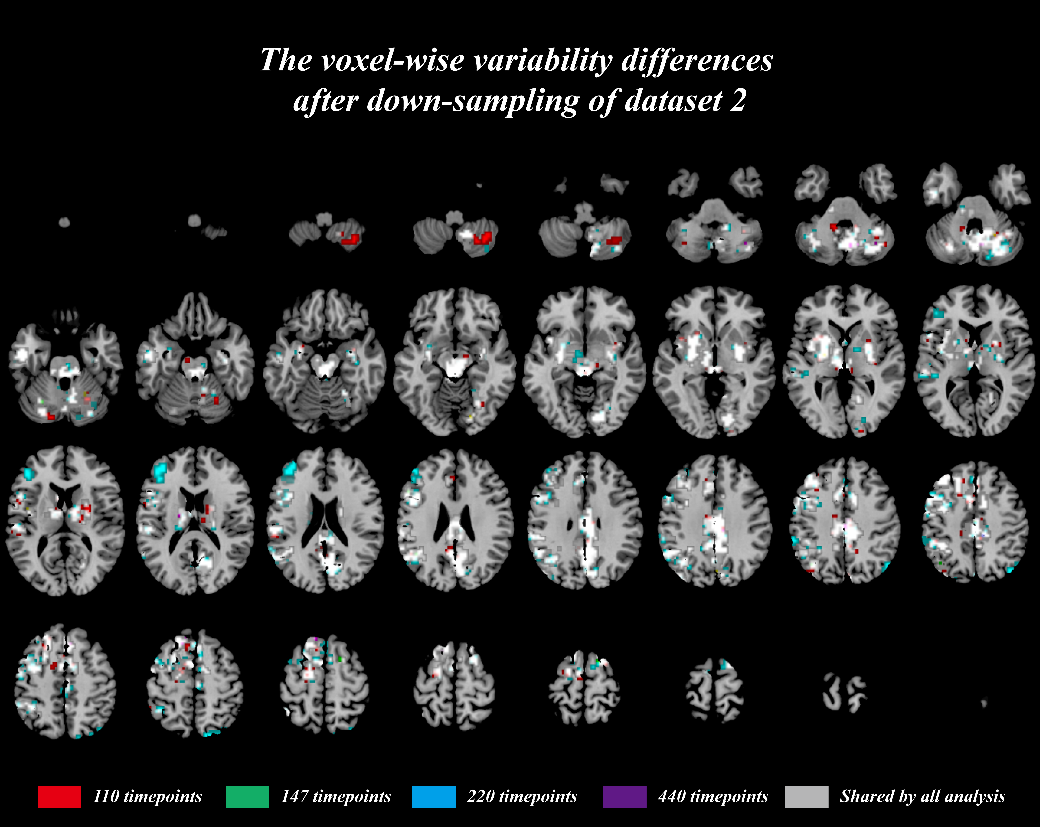


The voxel-wise signal variability differences between CSM patients and HCs after down-sampling dataset2 (pick out one timepoint out of every two, three, four timepoints). The grey color showed the overlapped brain regions of all analyses.

**Sup-Figure 3.**


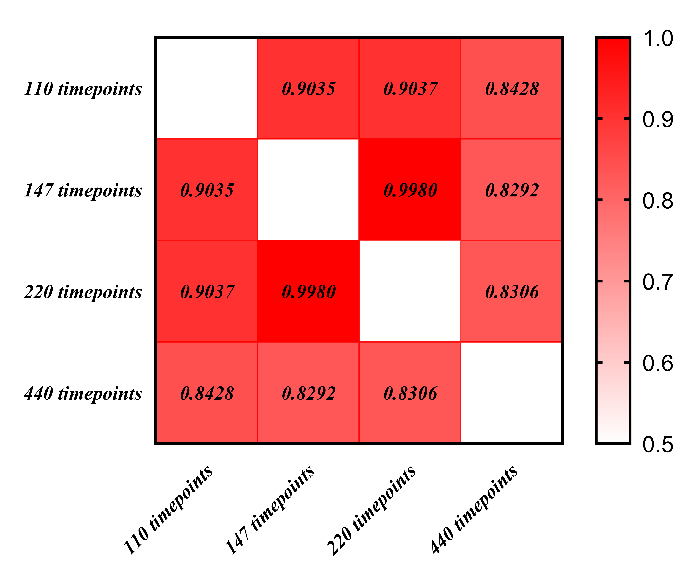


The dice correlation matrix of the binarized T maps (FWE corrected at cluster level; voxel-level p < 0.001, cluster-level p < 0.05). The greater correlation coefficient, the larger the overlapped between two maps.

**Sup-Figure 4.**


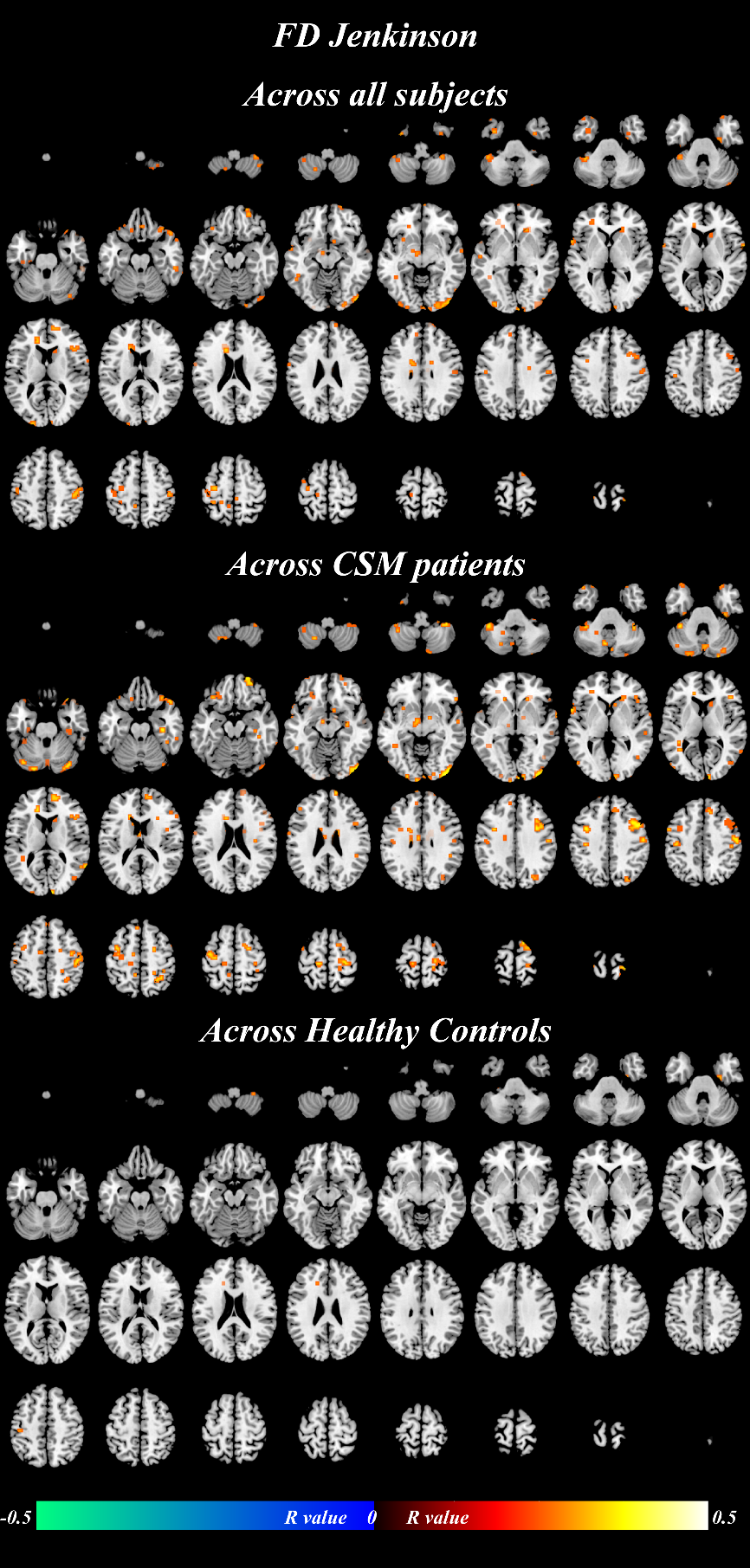


The voxel-wise correlation between voxel-wise signal variability and FD Jenkinson value (p-value < 0.001; uncorrected). No significant correlation was observed after cluster-level FWE or FDR correction.

**Sup-Figure 5.**


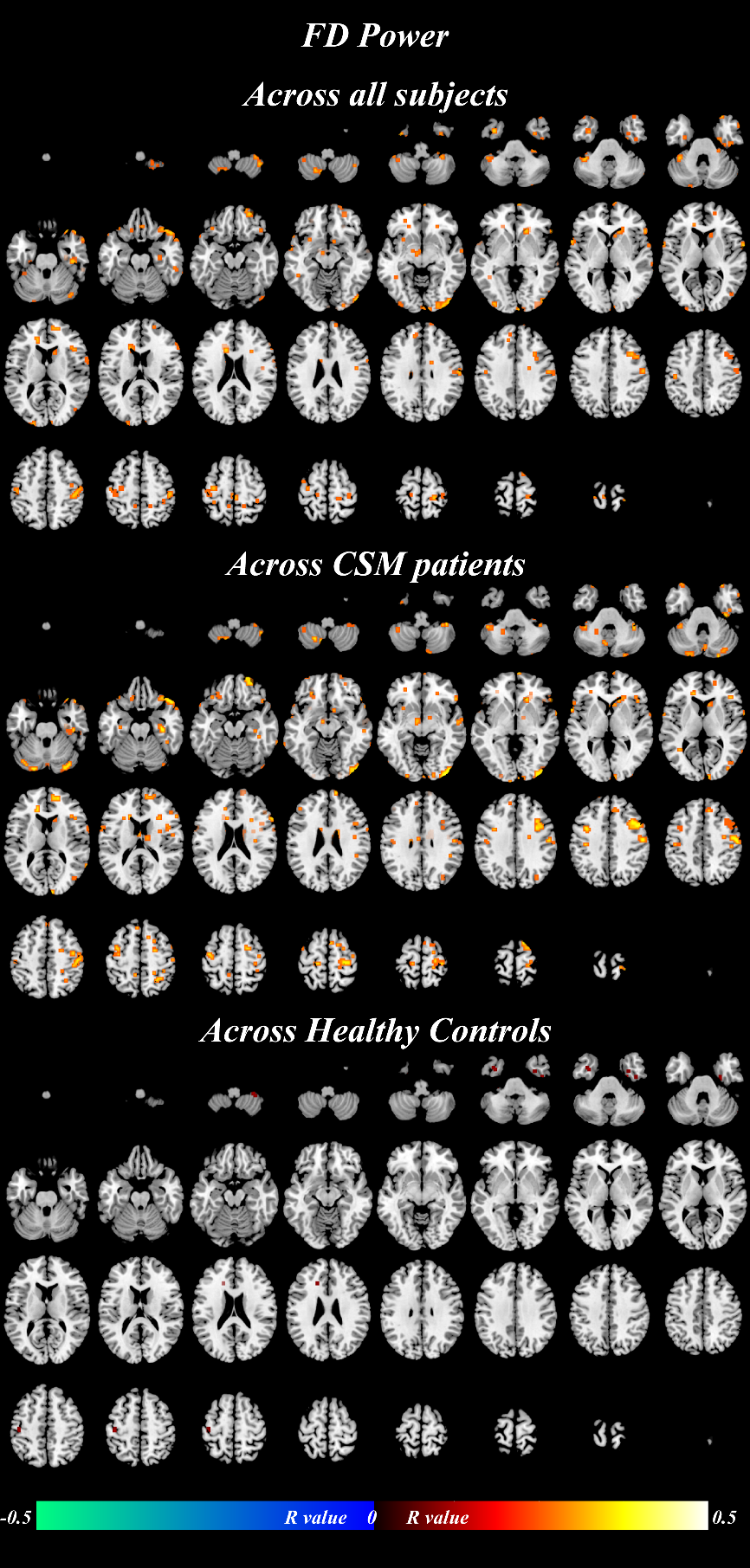


The voxel-wise correlation between voxel-wise signal variability and FD Power value (p-value < 0.001; uncorrected). No significant correlation was observed after cluster-level FWE or FDR correction.

**Sup-Figure 6.**


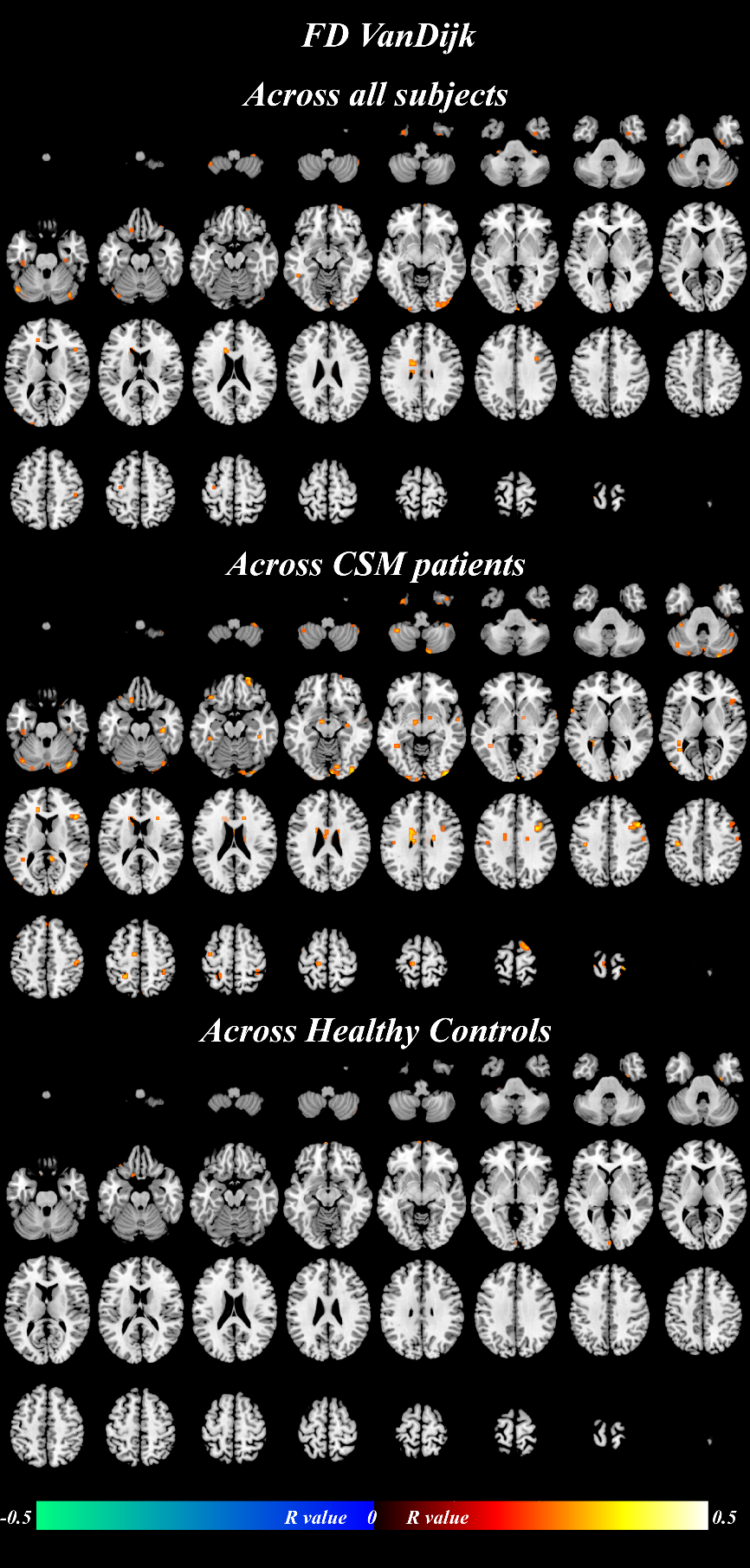


The voxel-wise correlation between voxel-wise signal variability and FD VanDijk value (p-value < 0.001; uncorrected). No significant correlation was observed after cluster-level FWE or FDR correction.

**Sup-Figure 7.
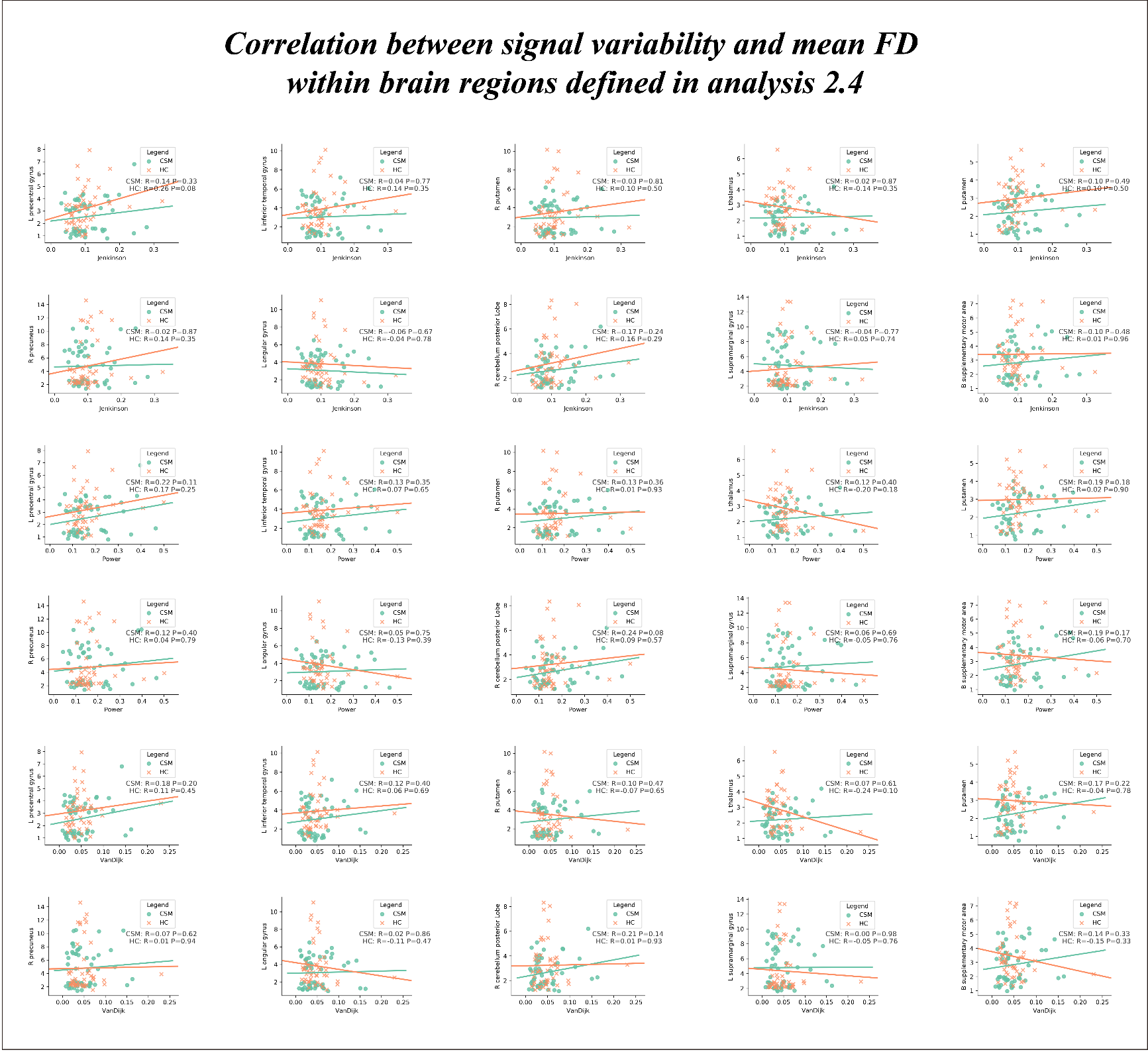
**

The correlation between FD value (i.e. FD Jenkinson, FD Power, FD VanDijk) and mean signal variability within brain regions reported in table 2. No significant correlation was observed (all p-values > 0.05).

**Sup-Figure 8.**

**
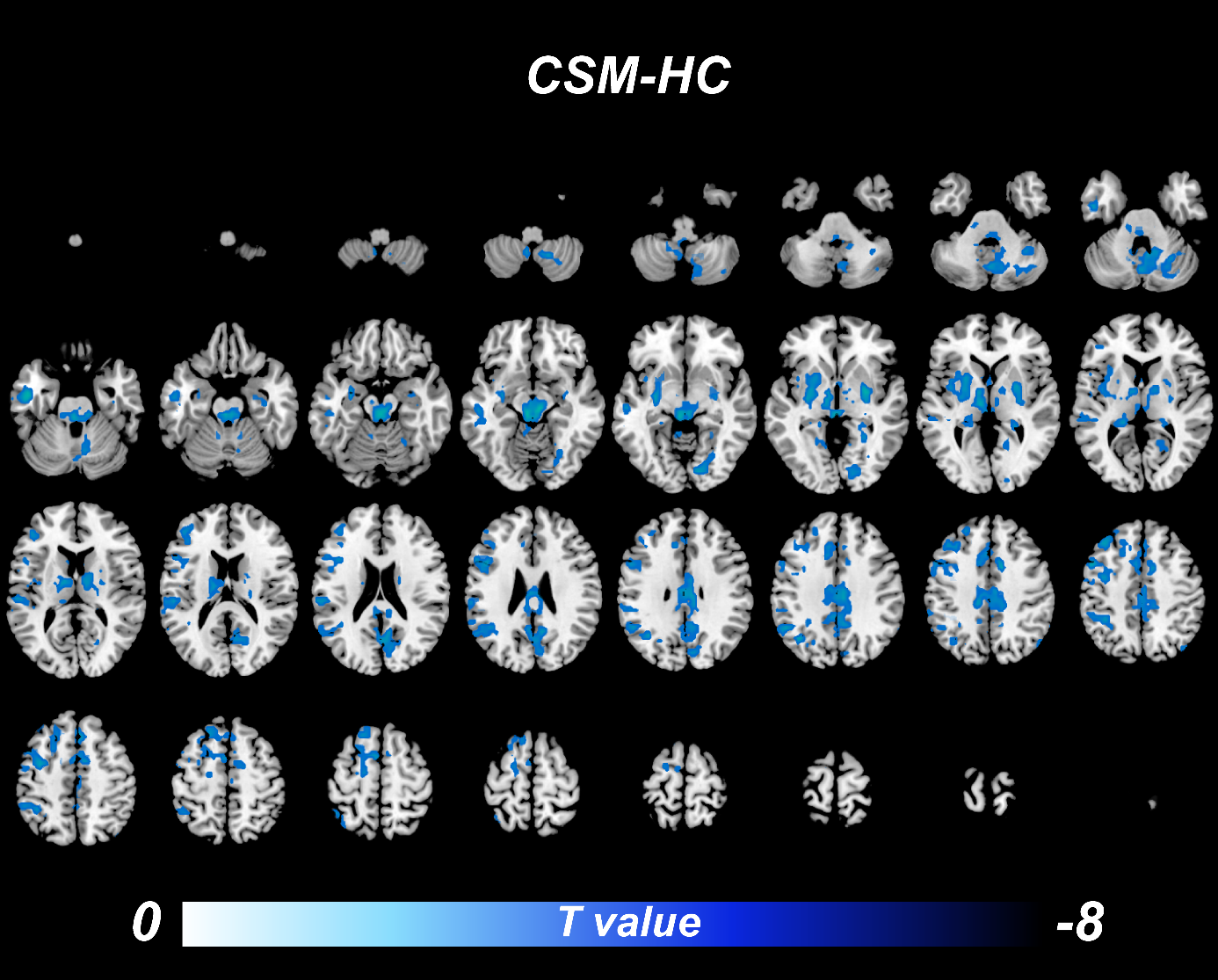
**

The voxel-wise signal variability differences between CSM patients HCs after regress out mean, max median FD Jenkinson value and six head-motion parameters.

**Sup-Figure 9.**

**
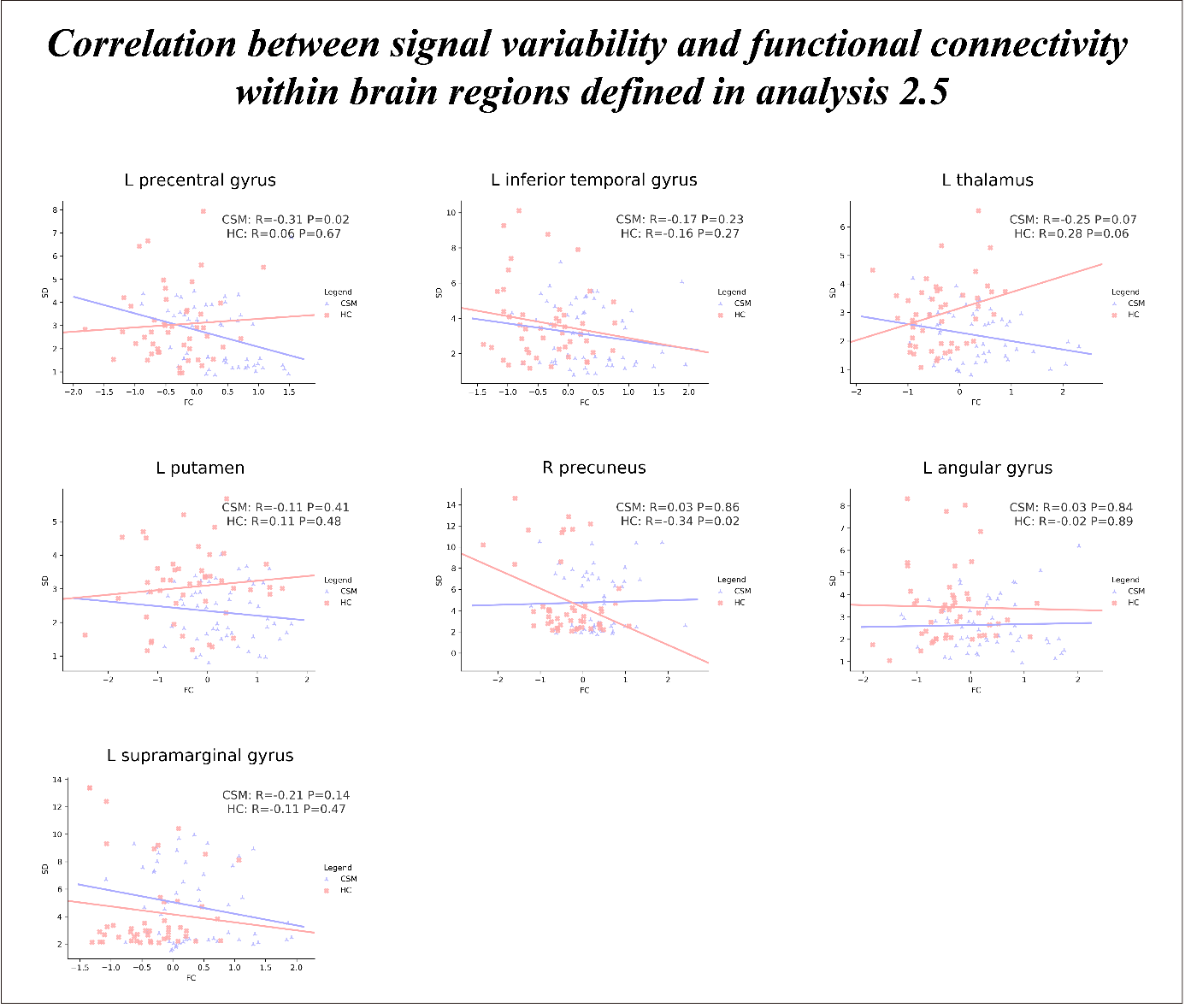
**

The correlation between altered signal variability and altered FCs within brain regions reported in table 4.

**Sup-Figure 10.**

**
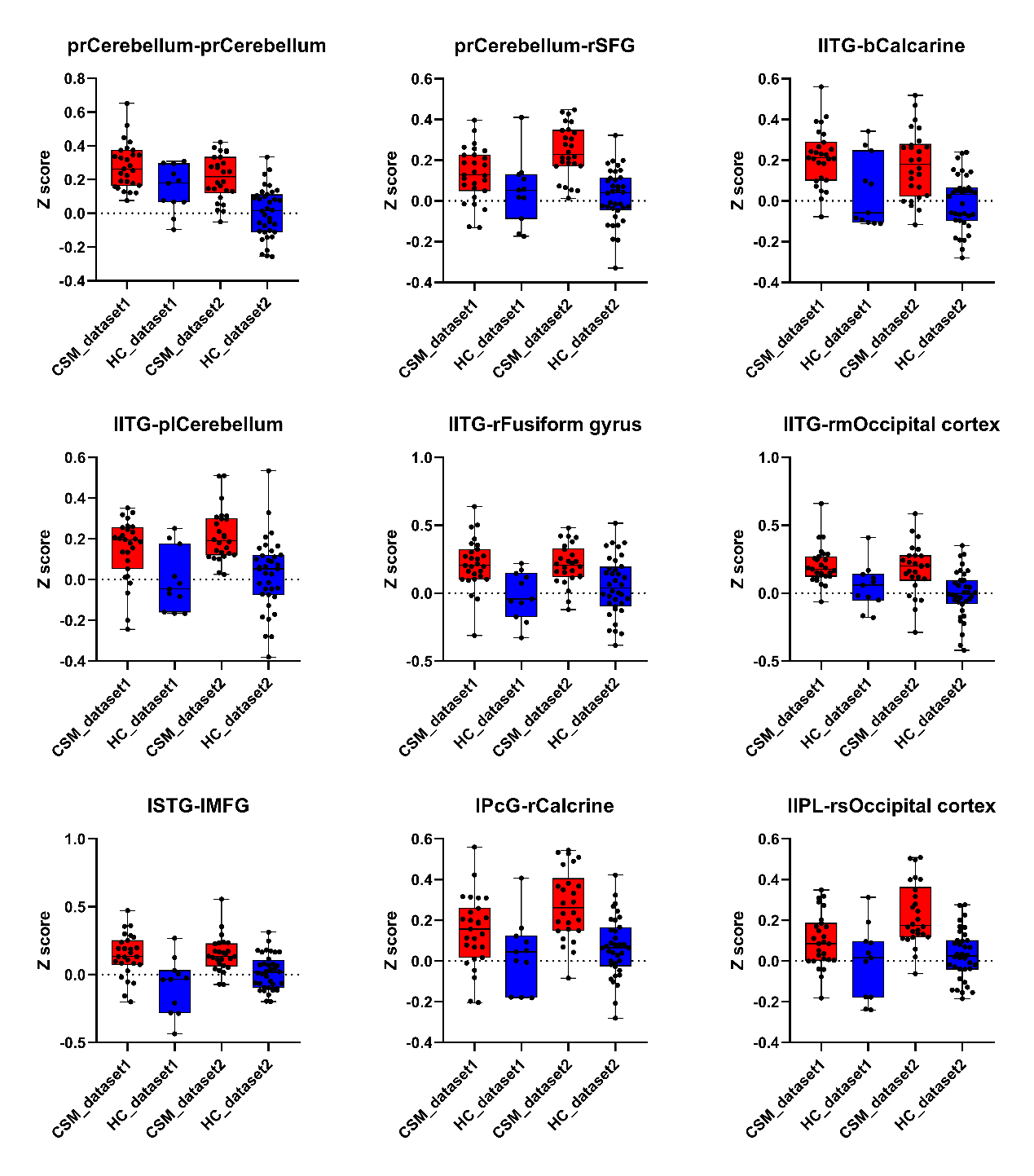
**

The FC differences between CSM patients and Healthy Controls within each dataset. Significant differences for FC were observed between CSM patients and HCs (all p-value < 0.001). prCerebellum: posterior right cerebellum lobe; rSFG: right superior frontal gyrus; lITG: left inferior temporal gyrus; bCalcarine: bilateral calcarine cortices; plCerebellum: posterior left cerebellum lobe; rFusiform gyrus: right fusiform gyrus; rmOccipital cortex: right middle occipital cortex; lSTG: left superior temporal gyrus; lMFG: left middle frontal gyrus; lPcG: left precentral gyrus; lIPL: left inferior parietal lobule; rsOccipital cortex: right superior occipital cortex.

**Sup-Figure 11.**

**
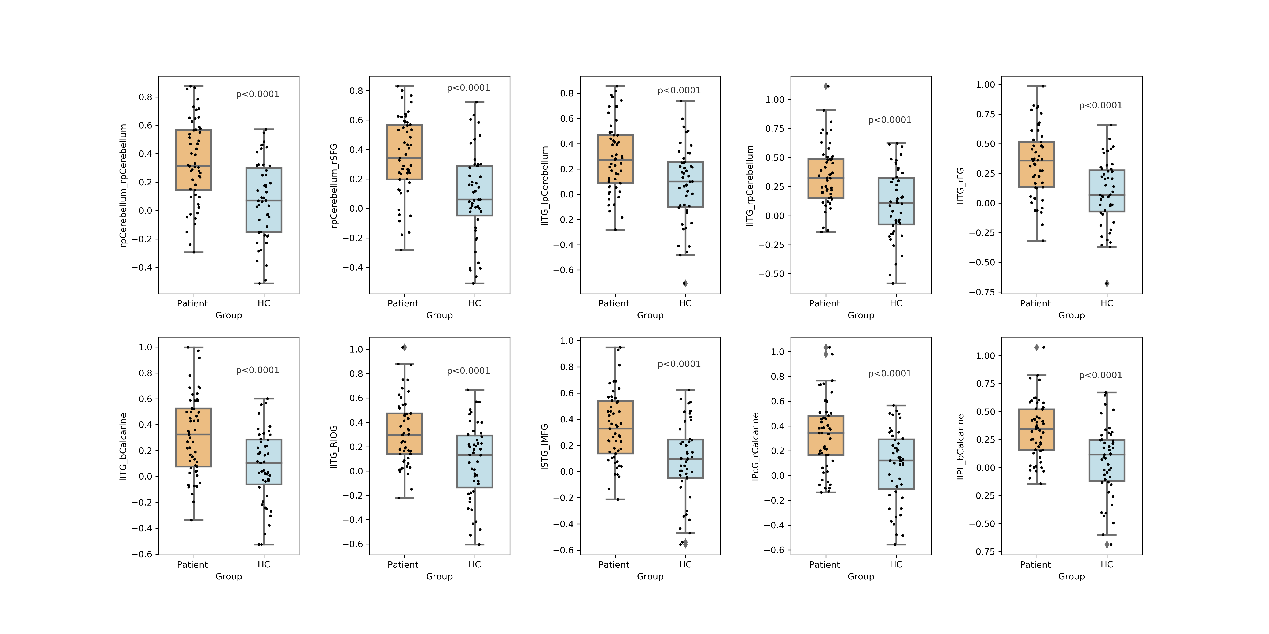
**

The seed-based functional connectivity (FC) differences between CSM patients and HC. Yellow: CSM patients; Blue: Healthy Controls.

**Sup-Figure 12.
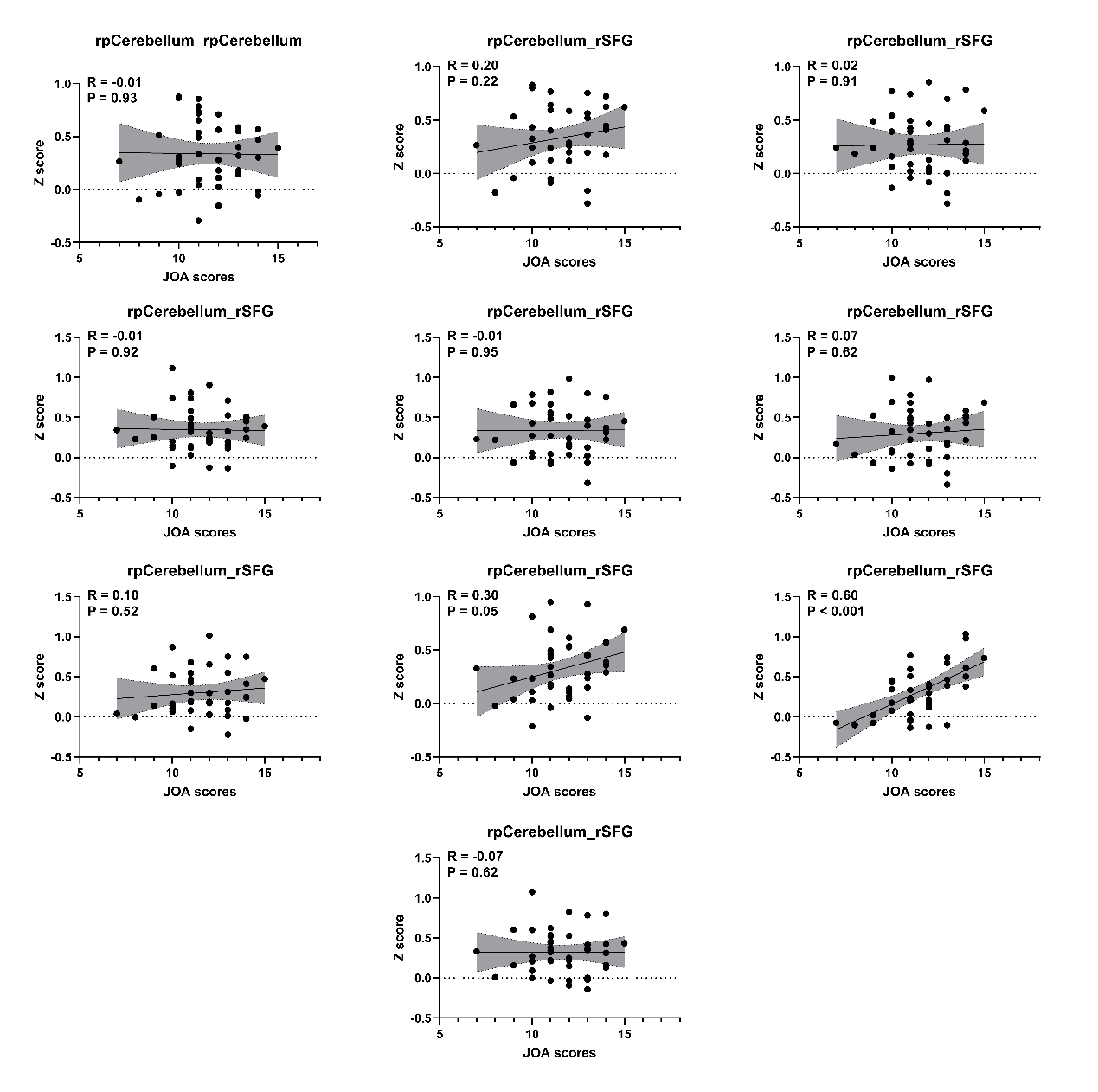
**The scatter plots of the correlation between altered FCs and JOA scores. prCerebellum: posterior right cerebellum lobe; rSFG: right superior frontal gyrus; lITG: left inferior temporal gyrus; bCalcarine: bilateral calcarine cortices; plCerebellum: posterior left cerebellum lobe; rFusiform gyrus: right fusiform gyrus; rmOccipital cortex: right middle occipital cortex; lSTG: left superior temporal gyrus; lMFG: left middle frontal gyrus; lPcG: left precentral gyrus; lIPL: left inferior parietal lobule; rsOccipital cortex: right superior occipital cortex.

**Sup-Figure 13.**

**
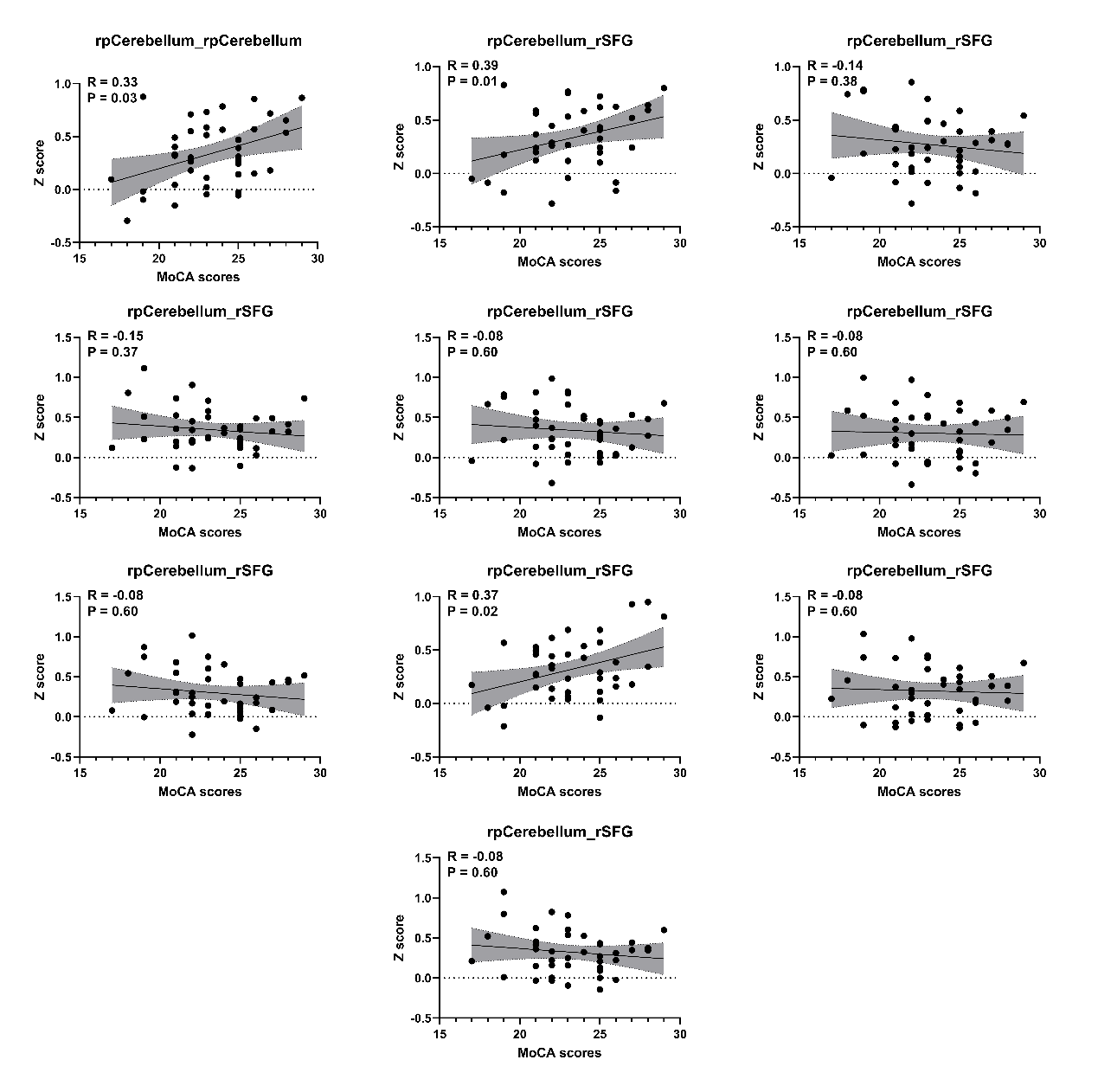
**The scatter plots of the correlation between altered FCs and MoCA scores. prCerebellum: posterior right cerebellum lobe; rSFG: right superior frontal gyrus; lITG: left inferior temporal gyrus; bCalcarine: bilateral calcarine cortices; plCerebellum: posterior left cerebellum lobe; rFusiform gyrus: right fusiform gyrus; rmOccipital cortex: right middle occipital cortex; lSTG: left superior temporal gyrus; lMFG: left middle frontal gyrus; lPcG: left precentral gyrus; lIPL: left inferior parietal lobule; rsOccipital cortex: right superior occipital cortex.

**Sup-Figure 14.**

**
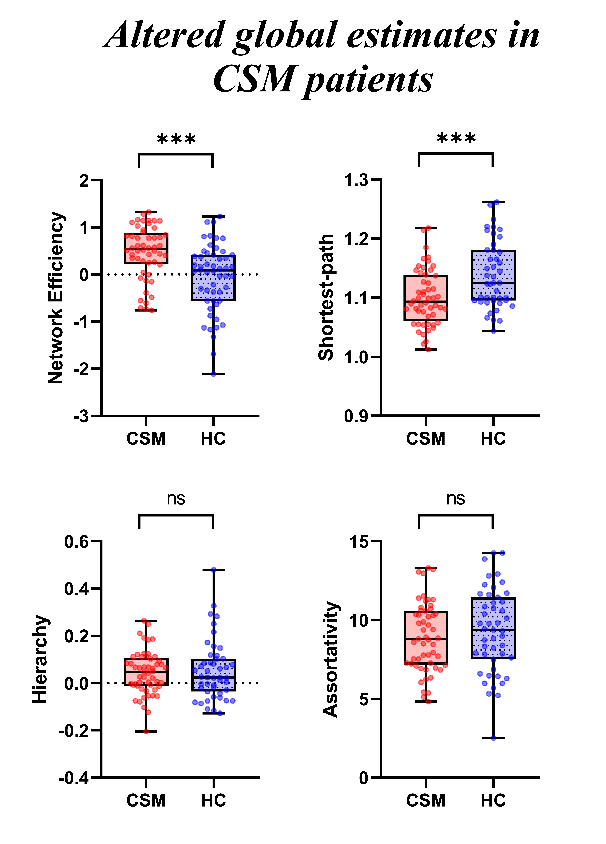
**

Box plots of differences for global extimates between CSM patients and healthy controls.

**Sup-Figure 15.**


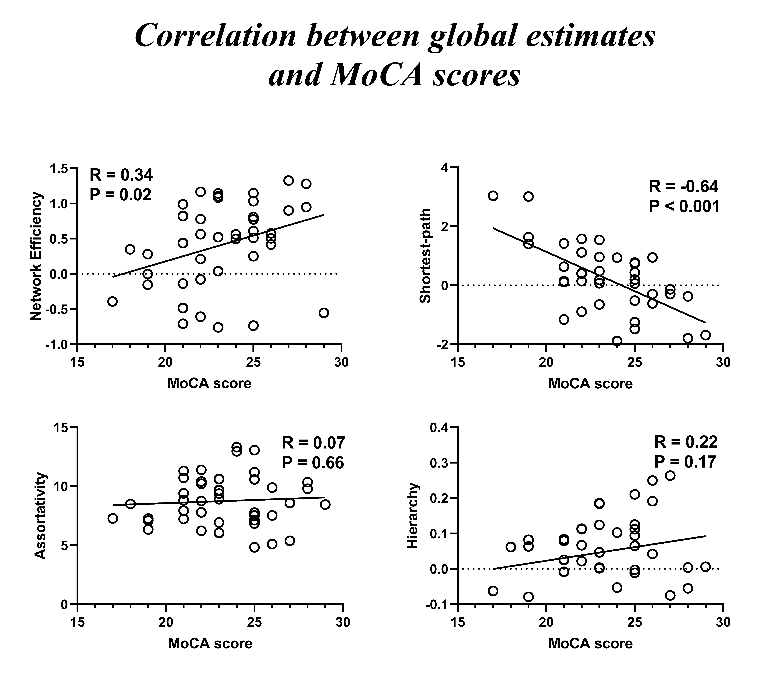


Scatter plots of correlations between global extimates and clinical measures.

**Sup-table 1.**

Regions of significant BOLD signal variability difference between CSM patients and HCs added mean FD Jenkinson, max FD Jenkinson, median FD Jenkinson and six head-motion parameters as covarites.

| **Brain Regions** | **Peak MNI (x, y, z)** | | | **Voxels** | **T value** |
| --- | --- | --- | --- | --- | --- |
| L thalamus | -6 | -21 | -12 | 152 | -6.07 |
| L precentral gyrus | -36 | -3 | 48 | 232 | -5.73 |
| R putamen/R pallidum | 27 | -6 | -3 | 119 | -5.66 |
| L inferior temporal gyrus | -48 | -6 | -27 | 75 | -5.64 |
| L superior temporal gyrus | -57 | -21 | 9 | 58 | -5.20 |
| R precuneus/R calcarine  /B posterior cingulate gyrus | 6 | -24 | 33 | 798 | -5.16 |
| L angular gyrus | -39 | -63 | 36 | 192 | -5.10 |
| R lingual gyrus | 15 | -84 | -9 | 106 | -4.96 |
| L supramarginal gyrus  /L superior temporal gyrus | -54 | -27 | 18 | 273 | -4.92 |
| L middle frontal gyrus | -36 | 30 | 45 | 209 | -4.88 |
| L angular gyrus | 63 | -51 | 45 | 74 | -4.69 |
| R hippocampus | 24 | -39 | 3 | 59 | -4.68 |

The clusters exhibited a significant difference between-group in CSM patients and HCs. L: left, R: right, B bilateral. The brain regions with peak MNI coordinates remain the same or only moved by few voxels were highlighted by red color.

**Sup-table 2.**

The correlation coefficients between the behavior scale scores and the BOLD signal variabilities within 8 mm peak group-difference spheres predefining.

|  | MoCA | JOA |
| --- | --- | --- |
| R cerebellum | 0.24 | 0.07 |
| L inferior temporal gyrus | -0.09 | -0.06 |
| L thalamus | 0.16 | -0.01 |
| L putamen | 0.22 | 0.25 |
| R putamen | **0.33*** | -0.04 |
| L superior temporal gyrus | -0.08 | 0.06 |
| R middle cingulate gyrus | 0.07 | **0.33*** |
| L precentral gyrus | -0.06 | **0.40*** |
| L inferior parietal lobule | **0.37*** | -0.04 |
| L superior frontal gyrus | -0.20 | -0.25 |

**Sup-table 3.**

Regions of significant functional connectivity differences between CSM patients and HCs.

| **Seeds** | **Regions** | **Peak MNI** | | | **Voxels** | **T** |
| --- | --- | --- | --- | --- | --- | --- |
| R Posterior cerebellum lobe | R cerebellum posterior Lobe | 23 | -84 | -48 | 82 | 4.69 |
|  | R superior frontal gyrus | -12 | 67 | 24 | 423 | 4.58 |
| L inferior temporal gyrus | L cerebellum posterior lobe | -39 | -51 | -45 | 88 | 4.65 |
|  | R cerebellum posterior lobe | 32 | -48 | -46 | 123 | 4.41 |
|  | R fusiform gyrus | -21 | -72 | -24 | 65 | 4.42 |
|  | B calcarine | 8 | -90 | 22 | 562 | 5.44 |
|  | R inferior occipital gyrus\ R middle occipital gyrus | -13 | -99 | -12 | 128 | 4.62 |
| L superior temporal gyrus | L middle frontal gyrus | -41 | 54 | 6 | 67 | 4.40 |
| L Precentral gyrus | R calcarine\R inferior occipital gyrus | 22 | -102 | -9 | 89 | 4.91 |
| L inferior parietal lobule | B calcarine\R superior occipital gyrus | 6 | -91 | 20 | 683 | 5.52 |

The clusters showed significant between-group functional connectivity differences between CSM patients and HCs. The brain regions with peak MNI coordinates remain the same or only moved by few voxels were highlighted by red color.
